# Supplementary material for: Defining cardiac cell populations and relative cellular composition of the early fetal human heart
Source: PLoS One. 2022 Nov 30;17(11):e0259477. doi: 10.1371/journal.pone.0259477 (PMC9710754; doi:10.1371/journal.pone.0259477)
Supplement: S1 Raw images — (PDF) [file pone.0259477.s001.pdf]

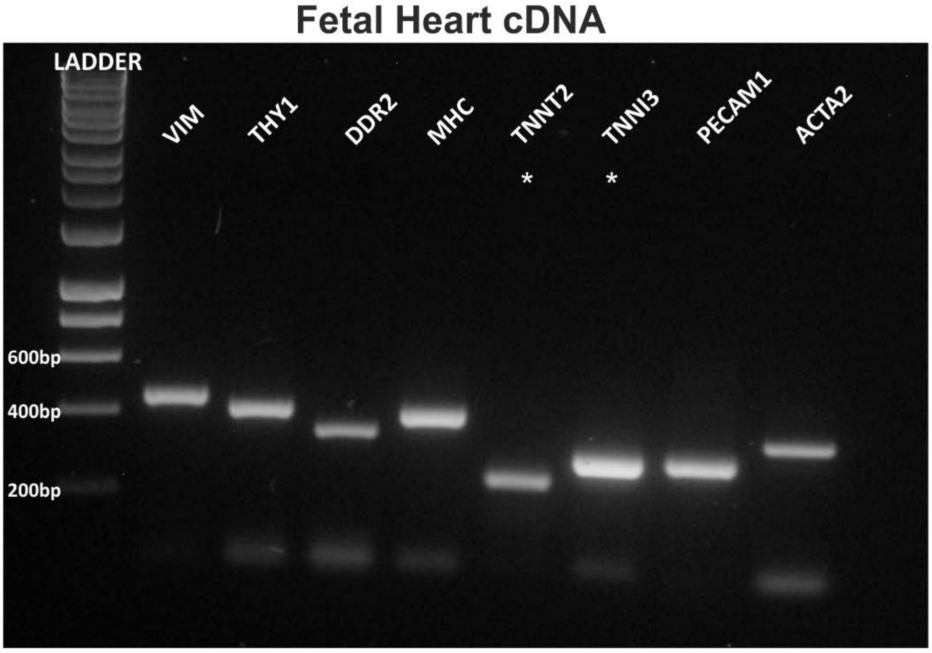

Agarose gel of reverse transcriptase PCR products from human fetal heart cDNA imaged using a UVP DigiDoc-It gel imager. Gel used to generate Figure 6A. The order of TNNT2 and TNNI3 bands (asterisked) were switched for Figure 6A

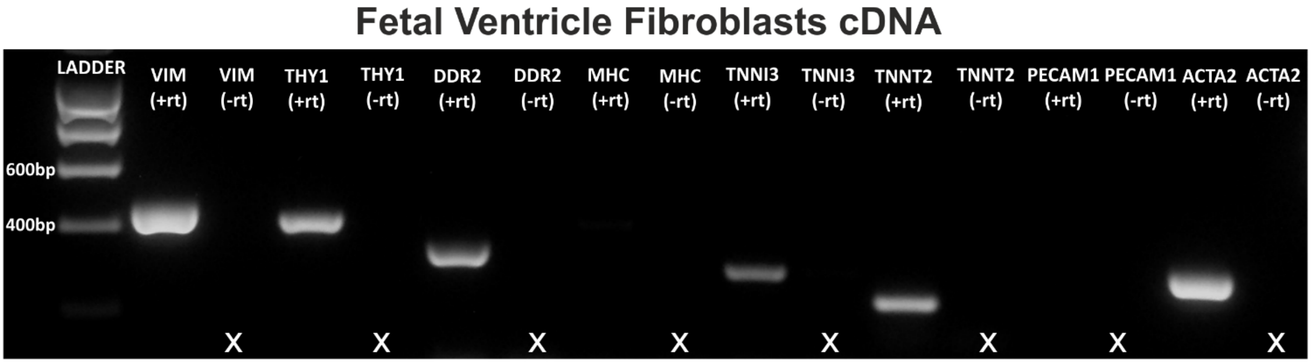

Original garose gel of reverse transcriptase PCR products from human fetal ventricle fibroblast cDNA imaged using a UVP DigiDoc-It gel imager. +rt refers to the use of reverse transcriptase in the generation of the cDNA; -rt refers to the absence of reverse transcriptase in the generation of the cDNA, used as a negative control. Gel used to generate Figure 6B. 'X' denotes lanes not included in final figure.
