# Supplementary figures and images for: Defining cardiac cell populations and relative cellular composition of the early fetal human heart
Source: PLoS One. 2022 Nov 30;17(11):e0259477. doi: 10.1371/journal.pone.0259477 (PMC9710754; doi:10.1371/journal.pone.0259477)

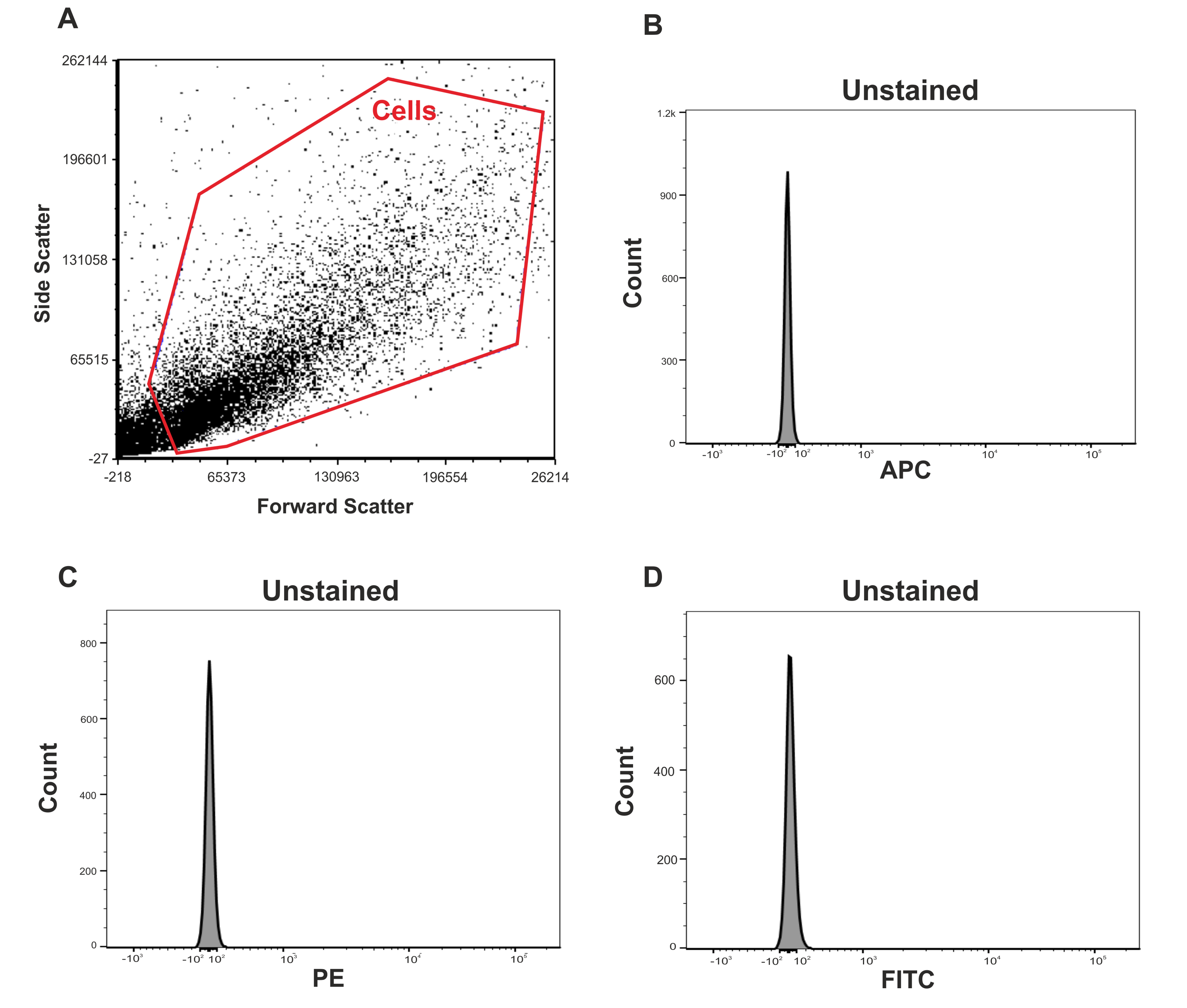

Supplement: S1 Fig — (A) Representative flow cytometry side scatter and forward scatter dot plot of fetal human cardiac cells showing gating of the cellular population for downstream analyses. (B) Representative flow cytometry histogram of unstained heart cells at the APC fluorophore wavelength (max excitation 650nm, max emission 661nm). (C) Representative flow cytometry histogram of unstained heart cells at the PE fluorophore wavelength (max excitation 566nm, max emission 574nm). (D) Representative flow cytometry histogram of unstained heart cells at the FITC fluorophore wavelength (max excitation 490nm, max emission 525nm). (TIF) [file pone.0259477.s002.tif]

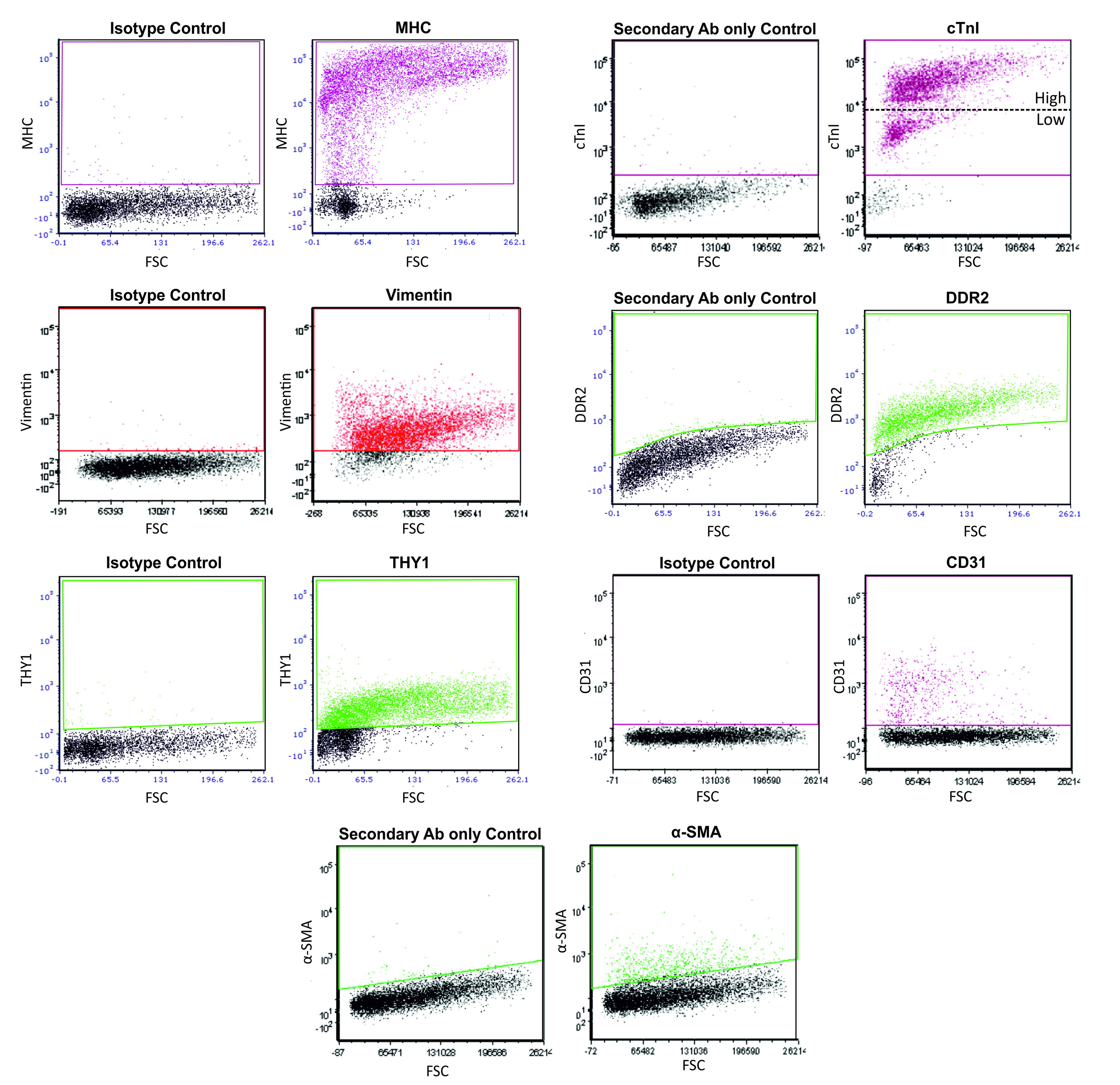

Supplement: S2 Fig — Dot plots with fluorescence on the Y axis and FSC on the X axis were used to determine positive populations. The distribution of the negative control population (isotype or secondary antibody only) was used to draw the positive gates. Cells expressing fluorescence levels above the negative controls were marked as positive. (TIF) [file pone.0259477.s003.tif]

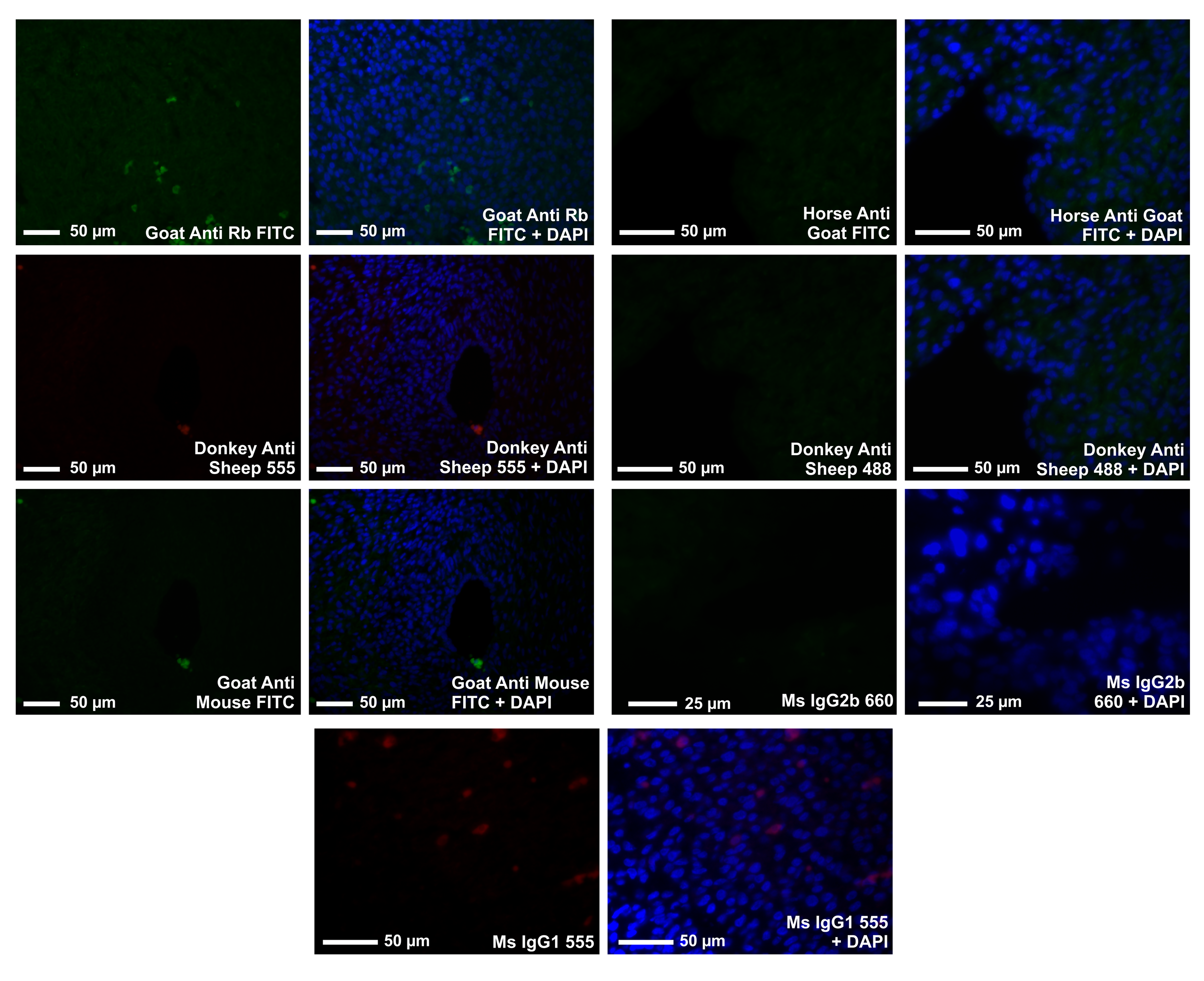

Supplement: S3 Fig — (TIF) [file pone.0259477.s004.tif]

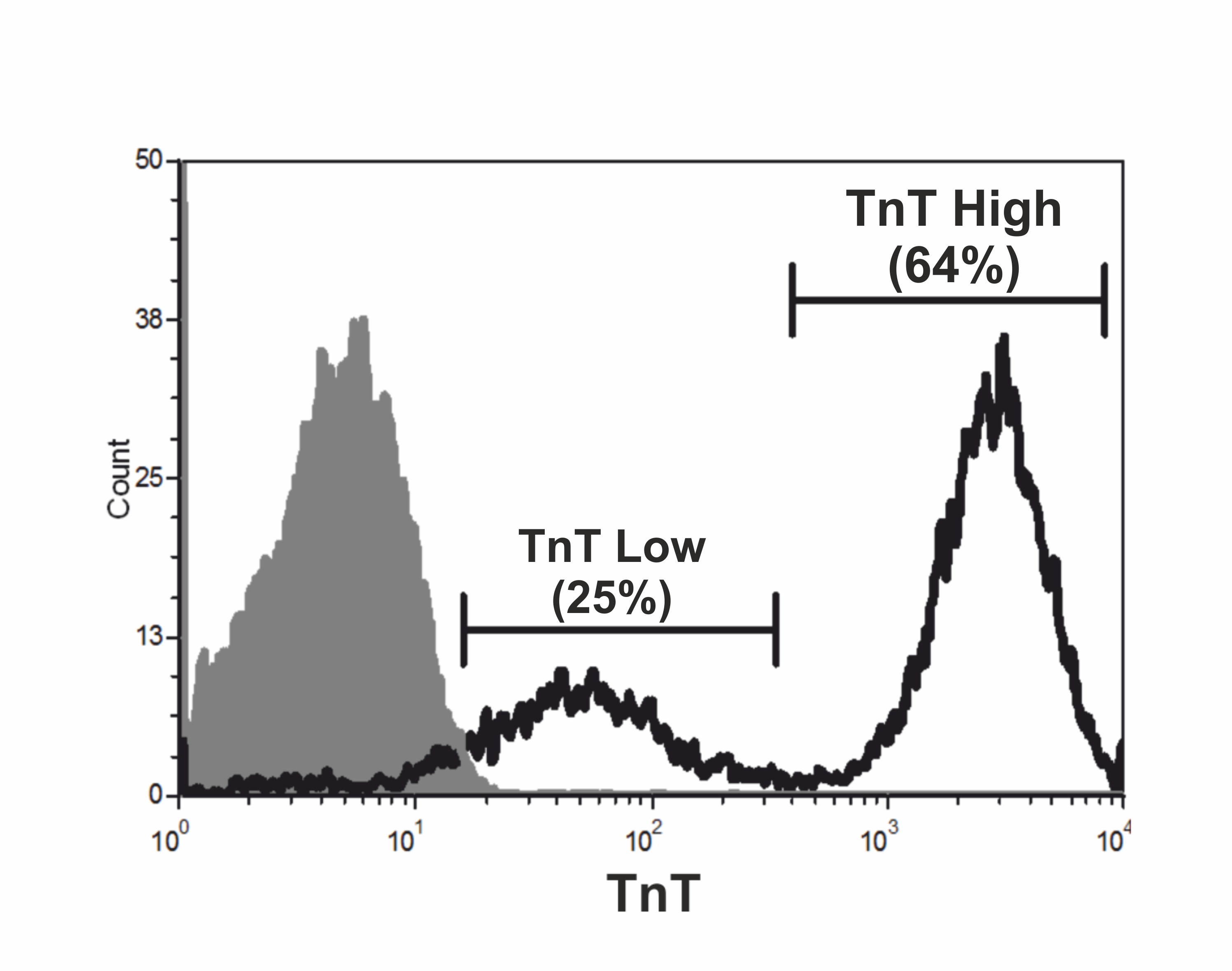

Supplement: S4 Fig — Grey histogram peaks represent antibody controls. (TIF) [file pone.0259477.s005.tif]

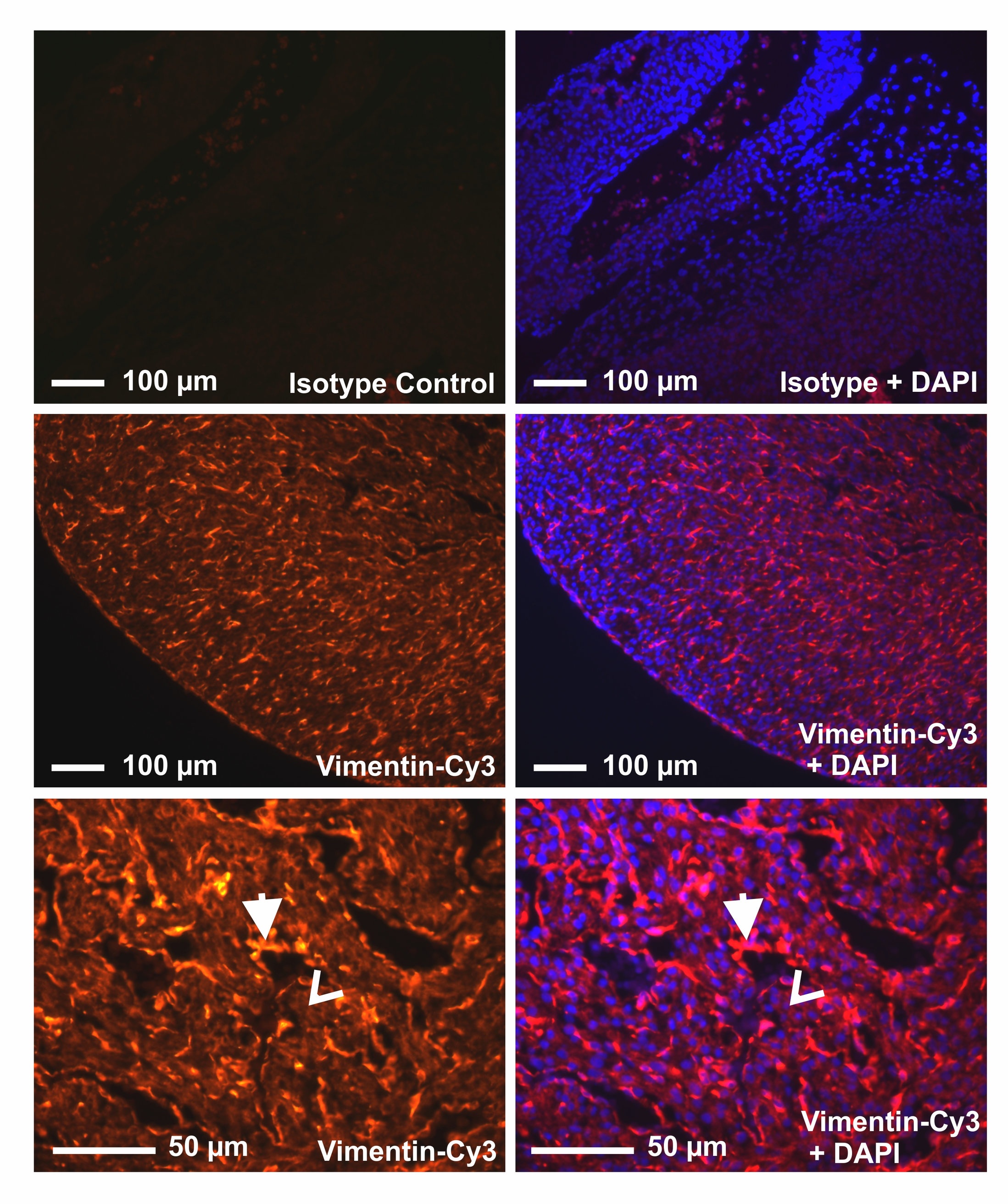

Supplement: S5 Fig — DAPI was used as a counter stain for cell nuclei. (TIF) [file pone.0259477.s006.tif]

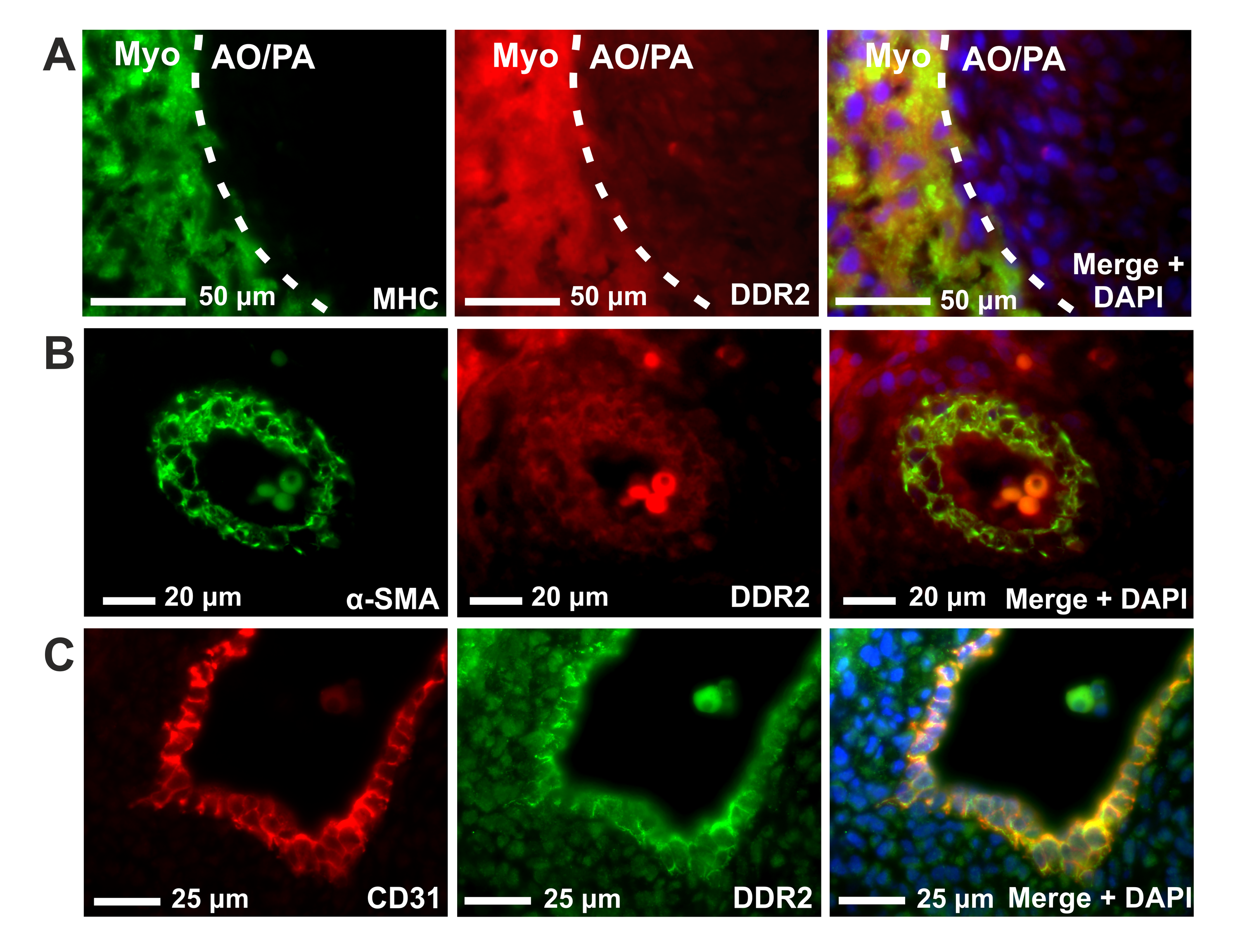

Supplement: S6 Fig — DDR2 dual immunohistochemistry of fetal human heart tissue: (A) DDR2 and MHC expression at the boundary (dotted line) of a blood vessel wall and the myocardium. (B) DDR2 and α-SMA expression in a cardiac blood vessel (C) DDR2 and CD31 expression in a cardiac blood vessel. Myo = myocardium. AO/PA = aorta/pulmonary artery. DAPI was used as a counter stain for cell nuclei. (TIF) [file pone.0259477.s007.tif]

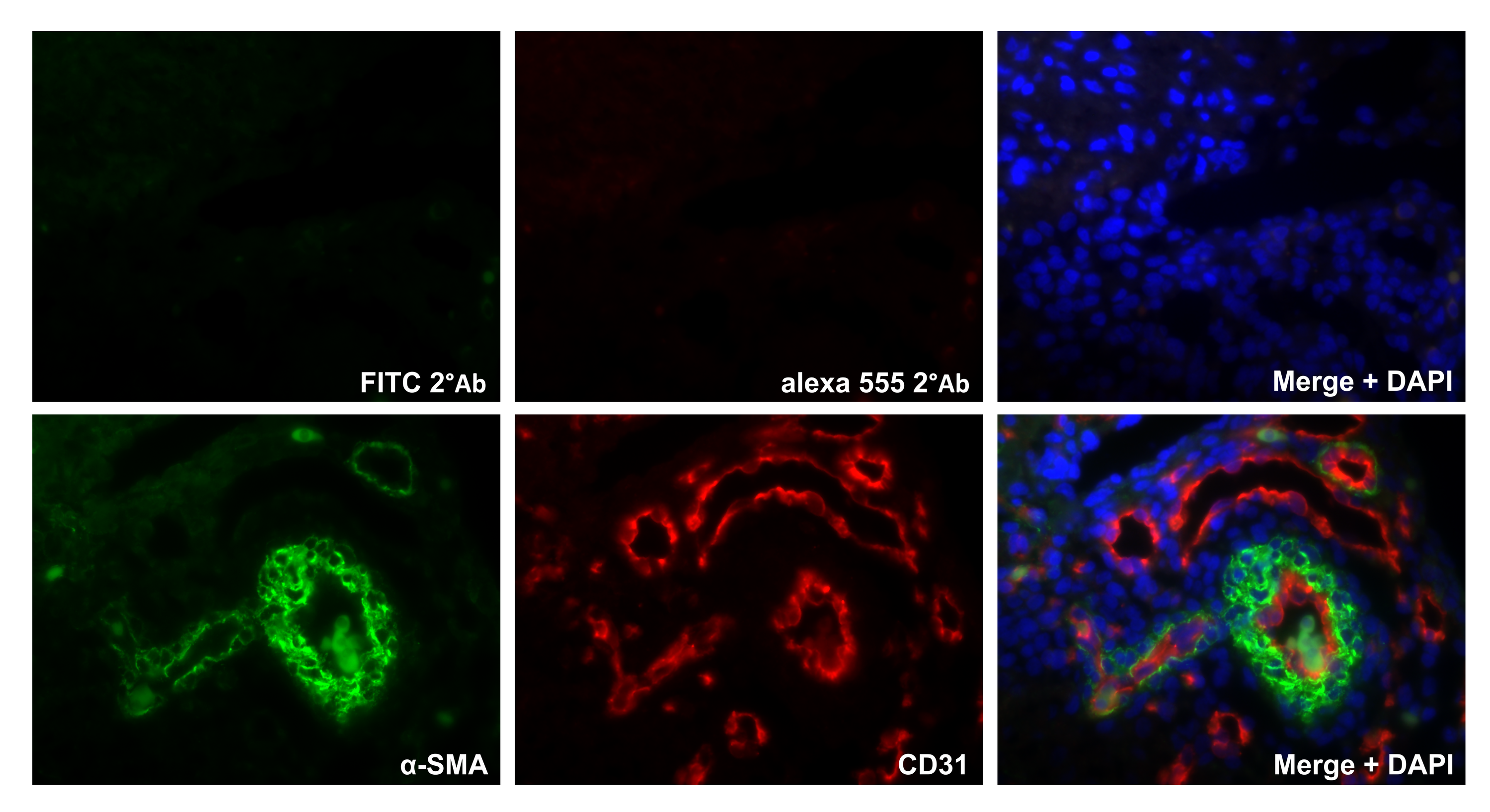

Supplement: S7 Fig — α-SMA expression is seen in the smooth muscle vessel lining and CD31 expression is seen in the endothelial inner lining of the vessel. DAPI was used as a counter stain for cell nuclei. (TIF) [file pone.0259477.s008.tif]
