# Supplementary material for: Defining cardiac cell populations and relative cellular composition of the early fetal human heart
Source: PLoS One. 2022 Nov 30;17(11):e0259477. doi: 10.1371/journal.pone.0259477 (PMC9710754; doi:10.1371/journal.pone.0259477)
Supplement: S1 Table — (DOCX) [file pone.0259477.s009.docx]

| **Name** | **Product Code** | **Applications** | **Concentration** | **Secondary antibody** | **Corresponding Control** |
| --- | --- | --- | --- | --- | --- |
| Thy1 | ProteinTech, 17641-1-AP | IHC | 1:100 | Goat anti-Rb FITC | 2° Ab only |
| Thy1-FITC | BD Biosciences, 555595 | FC | 1:100 | Directly conjugated | Ms IgG1-FITC (immunotools 21275513) |
| DDR2 | Santa Cruz, Sc-7555 | IHC, FC | FC: 1:50  IHC: 1:100 | Horse anti-Goat FITC | 2° Ab only |
| Troponin I | Abcam, Ab47003 | IHC, FC | FC: 1:100  IHC: 1:500 | Goat anti Rb Dylight 649  Goat anti-Rb FITC | 2° Ab only |
| Troponin T | Abcam, Ab8295 | FC | 1:100 | Goat anti Rb Dylight 649 | 2° Ab only |
| CD31 | R&D system, AF806 | IHC | 1:1000 | Donkey anti-Sheep alexa 555  Donkey anti-Sheep alexa 488 | 2° Ab only |
| CD31-APC | Immunotools, 21270316 | FC | 1:50 | Directly conjugated | Ms IgG1-APC (immunotools 21275516) |
| α-SMA | Leica, SMA-L-CE | IHC, FC | 1:100 | Goat anti Ms FITC | 2° Ab only |
| Vimentin-Cy3 | Sigma, C9080 | IHC, FC | FC: 1:100  IHC: 1:200 | Directly conjugated | Ms IgG1 Alexa-555 (CellSignal 9641S) |
| Myosin Heavy Chain- eFluor 660 | ebioscience 50-6503-80 | IHC, FC | FC: 1:100  IHC: 1:200 | Directly conjugated | Ms IgG2b eFluor 660 (ebioscience, 50-4732-82) |
