# Supplementary material for: Defining cardiac cell populations and relative cellular composition of the early fetal human heart
Source: PLoS One. 2022 Nov 30;17(11):e0259477. doi: 10.1371/journal.pone.0259477 (PMC9710754; doi:10.1371/journal.pone.0259477)
Supplement: S2 Table — (DOCX) [file pone.0259477.s010.docx]

| **Gene** | **Primer Sequence (5’ to 3’)** | **Product Size** |
| --- | --- | --- |
| VIM (Vimentin) | F: GAGAACTTTGCCGTTGAAGC  R: TCTTGCGCTCCTGAAAAACT | 469 bp |
| THY-1 | F: ACCCGTGAGACAAAGAAGCA  R: CTCAAGGTTTGAGGGATTGG | 427 bp |
| DDR2 | F: TCCAGCTATATGCCGCTATC  R: CTATTTCCATCCAGCACCTG | 357 bp |
| MYH6 (α-MHC) | F: GTCATTGCTGAAACCGAGAATG  R: GCAAAGTACTGGATGACACGCT | 413 bp |
| TNNI3 (cTnI) | F: GCCTCGAGAAAATTGCAGCT  R: CCGCTTAAACTTGCCTCGAA | 297 BP |
| TNNT2 (cTnT) | F: ATGATGCATTTTGGGGGTTA  R: TGCTGCTTGAACTTCTCCTG | 221 bp |
| PECAM-1 (CD31) | F: TGCAGTGGTTATCATCGGAGT  R: GACAGCTTTCCGGACTTCAC | 286 bp |
| ACTA2 (αSMA) | F: CTGTTCCAGCCATCCTTCAT  R: TGATCCACATCTGCTGGAAG | 292 bp |
