# Supplementary material for: Defining cardiac cell populations and relative cellular composition of the early fetal human heart
Source: PLoS One. 2022 Nov 30;17(11):e0259477. doi: 10.1371/journal.pone.0259477 (PMC9710754; doi:10.1371/journal.pone.0259477)
Supplement: S3 Table — (DOCX) [file pone.0259477.s011.docx]

|  | **Fetal Age (PCW)** | **MHC** | **TnI** | **Vimentin** | **DDR2** | **Thy-1** | **CD31** | **α-SMA** |
| --- | --- | --- | --- | --- | --- | --- | --- | --- |
|  | 11 |  |  | 96 |  |  | 10 |  |
|  | 12 |  |  | 95 |  |  | 7 |  |
|  | 8 | 76 |  | 85 |  |  |  |  |
|  | 8 | 77 |  | 89 |  |  | 8 |  |
|  | 9 | 73 |  | 90 |  | 74 |  |  |
|  | 11 | 85 |  | 82 |  | 80 | 11 |  |
|  | 12 | 84 |  | 88 |  | 83 | 9 |  |
|  | 11 | 75 | 92 | 92 |  |  | 8 | 11 |
|  | 9 | 73 |  | 89 |  | 78 |  |  |
|  | 12 | 73 | 94 | 92 |  |  |  |  |
|  | 11 | 83 | 99 | 93 |  | 89 | 10 |  |
|  | 9 | 72 | 94 | 92 |  | 82 |  |  |
|  | 12 |  | 97 |  |  | 86 |  | 10 |
|  | 11 |  | 96 | 93 |  | 69 | 8 | 6 |
|  | 8 | 72 | 94 |  |  | 80 |  |  |
|  | 11 | 79 | 92 | 94 |  | 86 | 8 |  |
|  | 10 | 68 | 87 | 86 |  | 87 | 13 | 20 |
|  | 8 | 76 | 99 |  | 82 | 81 |  |  |
|  | 10 | 70 | 85 | 81 |  | 75 |  |  |
|  | 12 | 73 | 88 | 88 |  | 79 | 7 | 13 |
|  | 8 | 72 | 84 |  |  |  |  | 7 |
|  | 10 | 74 |  |  |  |  |  |  |
|  | 12 | 78 | 99 | 87.9 | 82 | 89 | 9 | 18 |
|  | 11 | 81 |  |  | 86 |  |  |  |
|  | 8 | 66 |  |  | 83 |  |  |  |
| **Average** | **10** | **75** | **93** | **90** | **83** | **81** | **9** | **12** |
| **N** | 25 | 21 | 14 | 18 | 4 | 15 | 12 | 7 |
| **StDev** | 1.5 | 5.0 | 5.3 | 4.3 | 1.8 | 5.7 | 1.8 | 5.2 |
| **SEM** | 0.3 | 1.1 | 1.4 | 1.0 | 0.9 | 1.5 | 0.5 | 2.0 |
