# Supplementary material for: Defining cardiac cell populations and relative cellular composition of the early fetal human heart
Source: PLoS One. 2022 Nov 30;17(11):e0259477. doi: 10.1371/journal.pone.0259477 (PMC9710754; doi:10.1371/journal.pone.0259477)
Supplement: Attachment — Submitted filename: Response to Reviewers, Dewing et al 2022.docx [file pone.0259477.s012.docx]

**PloS One submission:**

***Defining Cardiac Cell Populations and Relative Cellular Composition of the Early Fetal Human Heart***

**α-SMA**

**DDR2**

**Response to reviewers**

**Reviewer 1:**

**Issue 1:**

We greatly appreciate the reviewers highlighting the issues with the methods section and the lack of clarity with regards to the antibodies used and the flow cytometry protocol. We now provide a table displaying the antibodies used for each application and their appropriate controls (Supplementary table 1). We have also updated the methods to include a more comprehensive protocol of flow cytometry and immunohistochemistry used in the study. Where possible, we used directly conjugated antibodies for flow cytometry analysis. The controls for such antibodies are isotype antibodies of the same antibody class, conjugated to the same fluorophore. For non-conjugated primary antibodies, we used a ‘secondary antibody only’ control. For each flow experiment, an ‘unstained’ cell sample was also used, and we have shown a representative histogram plot of this data in figure 2. Where possible, we used the same antibodies for both flow cytometry and IHC, and while we were able to do this for α-SMA, DDR2, troponin I, myosin heavy chain and vimentin, it was not possible for CD31 and Thy-1, despite repeated attempts and multiple IHC antigen retrieval protocols. We were therefore required to use separate antibodies for these analyses.

**Issue 2:**

We have now added a supplementary figure showing representative images of negative controls for immunohistochemistry (Supplementary figure 2), showing either ‘secondary only controls’ or appropriate ‘isotype controls’ (as detailed in the new antibody table).

We appreciate the reviewers’ comments on antibody validation. We chose antibodies that were from reliable companies where validation is standard practice and where possible had been previously published in research articles. The reviewers have suggested controls including testing against transgenic mice and validation by western blotting. Although the hypothetical rationale for these suggestion is sound, such experiments are not necessarily practical. Specifically, the conjugated primary antibodies used in our study were optimised for flow cytometry and fluorescence IHC, which means they may not be suitable for validation by western blot.

Below are examples of published articles that have used flow cytometry/IHC to investigate cardiac cells that did not include the validation levels being suggested by the reviewers.

- *Banerjee et al., (2007)* [*Determination of cell types and numbers during cardiac development in the neonatal and adult rat and mouse*](https://journals.physiology.org/doi/abs/10.1152/ajpheart.00514.2007)*. 293:3, H1883-H1891. DOI:* [*https://doi.org/10.1152/ajpheart.00514.2007*](https://doi.org/10.1152/ajpheart.00514.2007)
- *Mara, et al., 2019. Identification and Isolation of Cardiac Fibroblasts From the Adult Mouse Heart Using Two-Color Flow Cytometry. Frontiers in Cardiovascular Medicine. 6. DOI: 10.3389/fcvm.2019.00105*
- *Rupert et al., (2020) Human Cardiac Fibroblast Number and Activation State Modulate Electromechanical Function of hiPSC-Cardiomyocytes in Engineered Myocardium. DOI:* [*https://doi.org/10.1155/2020/9363809*](https://doi.org/10.1155/2020/9363809)
- *Kanisicak  et al., (2016) Genetic lineage tracing defines myofibroblast origin and function in the injured heart. Nat Commun 7, 12260. DOI: https://doi.org/10.1038/ncomms12260*
- *Pinto et al., (2016). Revisiting Cardiac Cellular Composition.* 118(3):400-9. *DOI:* [*10.1161/CIRCRESAHA.115.307778*](https://doi.org/10.1161/circresaha.115.307778)

In order to help validate the antibodies and confirm they detected the proteins of interest, we used IHC to investigate the staining pattern and localisation of the antibodies in human fetal heart tissue. The sarcomeric staining observed for both MHC and troponin I (TnI) antibodies, and the vascular staining for α-SMA and CD31, are the expected antibody staining patterns. Regarding the reviewer’s comment that we should have done double labelling to show there was no overlapping of α-SMA and CD31, we now show these data: please see the image below showing distinct patterns of expression for these antibodies, which we have now added as Supplementary Fig 6.

*Supplementary Figure 6. Dual staining of α-SMA and CD31. (A) Secondary antibody only controls (B) α-SMA expression in smooth muscle lining of the vessel and CD31 expression in the endothelial inner lining of the vessel.*

The key findings of our study relate to MHC, cTnT and vimentin expression levels in the fetal heart and the antibodies for these have been well cited and characterised by previously published work:

**MHC (ebioscience 50-6503-80): 11 citations including:**

- Martinez-Fernadez *et al.,* 2014. ***iPS cell-derived cardiogenicity is hindered by sustained integration of reprogramming transgenes***. DOI: 10.1161/CIRCGENETICS.113.000298
- Martinez-Lagunaz *et al.,* 2020. **In vivo detection of programmed cell death during mouse heart development.** DOI: 10.1038/s41418-019-0426-2
- Ifkovits *et al., 2014* ***Inhibition of TGFβ signaling increases direct conversion of fibroblasts to induced cardiomyocytes.*** DOI: 10.1371/journal.pone.0089678

**cTnI (Abcam, ab45932): 71 citations including:**

- [Vigil-Garcia](https://pubmed.ncbi.nlm.nih.gov/?term=Vigil-Garcia+M&cauthor_id=32717063) *et al.,* 2021. ***Gene expression profiling of hypertrophic cardiomyocytes identifies new players in pathological remodelling.*** DOI: [10.1093/cvr/cvaa233](https://doi.org/10.1093/cvr/cvaa233)
- [Gladka](https://pubmed.ncbi.nlm.nih.gov/?term=Gladka+MM&cauthor_id=33398012) *et al.,* 2021**. Cardiomyocytes stimulate angiogenesis after ischemic injury in a ZEB2-dependent manner**. DOI: [10.1038/s41467-020-20361-3](https://doi.org/10.1038/s41467-020-20361-3)
- [Ozcebe](https://pubmed.ncbi.nlm.nih.gov/?term=Ozcebe+SG&cauthor_id=33296796) et al., 2021. ***Effect of cellular and ECM aging on human iPSC-derived cardiomyocyte performance, maturity and senescence.*** DOI: [10.1016/j.biomaterials.2020.120554](https://doi.org/10.1016/j.biomaterials.2020.120554)

**Vimentin (Sigma, C9080): 40 citations including:**

- Chiu et al., 2011. [**Biphasic Electrical Field Stimulation Aids in Tissue Engineering of Multicell-Type Cardiac Organoids**](https://www.liebertpub.com/doi/full/10.1089/ten.tea.2007.0244)**.** <https://doi.org/10.1089/ten.tea.2007.0244>
- [Christoforou](https://pubmed.ncbi.nlm.nih.gov/?term=Christoforou+N&cauthor_id=28071742) et al., 2017. **Core Transcription Factors, MicroRNAs, and Small Molecules Drive Transdifferentiation of Human Fibroblasts Towards The Cardiac Cell Lineage**. DOI: [10.1038/srep40285](https://doi.org/10.1038/srep40285)
- Ze-Wei *et al*., 2018 **Bioengineering Cardiac Tissue Constructs With Adult Rat Cardiomyocytes.** DOI: 10.1097/MAT.0000000000000765

Fibroblasts are a less well-defined cell type than cardiomyocytes, endothelial and smooth muscle cells and have a less specific pattern of localisation in human heart tissue, which makes validating ‘so called’ fibroblast markers more difficult. However, we believe the positive expression of vimentin, DDR2 and Thy-1 in fibroblasts isolated from human heart tissue, analysed by IHC, flow cytometry and PCR, is sufficient to show these protein are expressed in these cells (and far exceeds the validation efforts of some of the previously published work mentioned above). We believe the lack of fibroblast specificity of these proteins is not a product of the antibodies used to detect them, but it is an issue of fibroblast markers reported to date, which we highlight in this study with the hope it will encourage others to consider how best to define fibroblasts and cardiomyocytes of the early developing heart.

The method of explant migration used to isolate fibroblasts in this study is a standard technique that has previously been used by many others (Ieda et al 2010. DOI:10.1016/j.cell.2010.07.002; Garbutt et al., 2020 [10.1016/j.xpro.2019.100010](https://doi.org/10.1016/j.xpro.2019.100010) ). The isolated cells had a characteristic fibroblast morphology and we were able to show by PCR analysis that expression of myosin heavy chain was absent from these isolated fibroblasts, indicating no cardiomyocyte contamination. We discuss this further under the comments for figure 6.

**Figure 2:**

**Issue 1:**

We have now added a more detailed description of the flow cytometry protocol used in this study to the materials and methods section. The design of figure 2 was based on the published figure by Banerjee et al 2007, in which they used flow cytometry to investigate cell type and number of the neonatal and adult rodent heart (see their figure below). In their study they used an unstained control to gate their positive cells. In our study, we display a representative image of an unstained cell population in figure 2A, but also used isotype and secondary only controls to gate our positive population, which we believe is a more accurate method of gating. All of our gating, except for myosin heavy chain (MHC), is positioned based on the control histogram. We chose to be more specific for MHC because of the narrow far peak and very low and broad first peak. We accept that the first smaller peak not included in the gate may represent a very small population of MHC positive cells, however, when this population is included in the gate, the overall number does not alter the percentage significantly. We believe the large peak represents the cardiomyocyte population.

*Figure taken from: Banerjee I, Fuseler JW, Price RL, Borg TK, Baudino TA. Determination of cell types and numbers during cardiac development in the neonatal and adult rat and mouse. American Journal of Physiology-Heart and Circulatory Physiology. 2007;293(3):H1883-H91*.

Please see below representative dot plots for MHC flow cytometry, not shown in the manuscript. The red gate includes all cells above the isotype control and the green gate selects the dense population of highly positive cells. Compared to the dot plots for Vimentin and TnI (Figures 3 A and C), which show two distinct populations, we did not feel that the dot plot for MHC exhibited the same distinct population distribution.

*Dot plots of myosin heavy chain (MHC) (Myosin Heavy Chain- eFluor 660) and the corresponding isotype control (Ms IgG2b eFluor 660 (ebioscience, 50-4732-82)*

**Issue 2:**

We have now added a more detailed description of the flow cytometry protocol to the materials and methods and added the antibody table to provide clarity.

**Issue 3:**

Ideally, we would have been able to use conjugated primary antibodies for all the flow cytometry analyses. However, we were limited by the commercial antibodies existing at the time and the fluorophores conjugated to these antibodies, as for dual flow cytometry we required different fluorophores excited by different lasers. The reason for the differences in the X-axes between the graphs is due to differences in the voltage setting of the different lasers due to the different fluorphores used (either conjugated to the primary antibodies or the secondary antibodies). The voltage is adjusted to position the cell population peak appropriately along the X-axis i.e. if there are very high levels of expression, the histogram peak may be too far to the right of the X-axis, and therefore the voltage is adjusted to bring the histogram to the left. Critically, the same voltage setting is applied to the negative control so you can compare expression between the test sample and the control sample. The information depicted in each of the graphs in figure 2 should therefore be interpreted stand-alone and does not require comparison between graphs. For clarity we have now amended the figure 2 legend to state: *Grey-coloured histograms represent the control sample: isotype controls for conjugated primary antibodies and secondary antibody only controls for non-conjugated primary antibodies.*

**Issue 4:**

We do not agree with the reviewer that the quality of the data varies considerably between different antigens. Flow cytometry for all antigens was conducted using the same protocol. The differences in the data represents the differences in the target antigens. The flow cytometry data published by Banerjee et al 2007 and shown on page 3 above depicts graphs analogous in quality and patterns to the graphs we have shown in figure 2. Specifically our flow cytometry graphs for CD31 and α-SMA are very similar to their published data.

**Issue 5:**

Please see our comments replying to issue 2 on the first page of the rebuttal discussing antibody validation.

**Figure 2a:**

We have now included the FSC x SSC plot as an additional supplementary figure (Supplementary Fig 1)

**Figure 2b-d:**

Please see our comments regarding the gating of MHC in our reply to figure 2, issue 1 on the previous page.

**Figure 2g:**

We are grateful this has been pointed out. When we re-examined the flow cytometry data it showed that the gating of CD31 was in fact measured from the start of the smaller high intensity peak and this illustrative error has now been amended. To be clear, the gate drawn for this figure is meant to act as a representative of the actual gating used to source the data and therefore the data itself is not affected.

**Figure 2h:**

The shift of the peak for α-SMA to the right of the control is consistent with the data published by Banerjee et al., 2007, shown on the previous page.

**Fig. 3**

**3a-e:**

The aim of figure 3 is to show the dual populations of cardiac cells positive for vimentin (3a, b) and cTnI (3c, d). This is firstly demonstrated as a dot plot and then displayed as a histogram. The gating for all flow cytometry data in this manuscript was drawn based on the dot plots, not the histograms, as this gives a more accurate representation of the distribution pattern of the negative cell populations. We have now added this information into the methods section. The distribution pattern of the cell populations in 3a and 3c were not perfectly perpendicular to the Y axis (fluorescence). This is likely due the variation in cell size between different cardiac populations, which can affect fluorescence levels. This is seen in other published flow cytometry data (Yen-Rei et al., 2016 <https://doi.org/10.1371/journal.pone.0150606>). The markers over the histogram peaks in fig 3b and 3c are not gates; they are there to highlight the two populations of cells, based on the gates from the dot plots.

The reviewer has questioned our interpretation of the flow cytometry data in figure 3. It is clear to see in figure 3e that the histogram of TnI in AO/PA tissue (red) has two peaks. The smallest peak on the left is at the same intensity as the secondary only (negative) control, indicative of this being the negative population. The intensity of the second peak is 100x greater than the first peak, and is at a similar intensity to the first peak of TnI in heart tissue. We believe the distribution of the peaks in Figure 3E is strong evidence that TnI is expressed at low levels in AO/PA. Furthermore, the immunohistochemistry images shown in figure 5A are further evidence of high and low TnI expression in the heart, as shown by the lower levels in the aorta/pulmonary artery relative to the myocardium.

Regarding the reviewer’s statement ‘*Troponin is used only by striated and not by smooth muscle’,* other evidence suggests that this may not be the case, with recent findings showing expression of troponin T in (non-striated) cardiac fibroblasts from 17 gestational week fetal cardiac tissue (Cui et al., (2019) doi: 10.1016/j.celrep.2019.01.079), consistent with what we have found. Furthermore, previous studies have shown the expression of other contractile proteins, including actin, myosin and tropomyosin in non-muscle cells, which suggests that cardiac troponin may also have non-muscle-specific isoforms, which we attempted to discuss in the discussion of our manuscript. We believe this is strong, impartial evidence supporting our findings with regard to troponin I in the human fetal heart.

**Figure 4:**

The table we have added to the manuscript showing all the antibodies used and the corresponding fluorophores will be able to clarify that appropriate fluorophores were used for dual flow cytometry. Specifically, whilst an APC secondary was used for TnI dual staining with vimentin (Cy3), for dual staining with MHC (efluor 660), an anti Rb FITC secondary was used for TnI. Unstained and single stained samples were used to set compensation controls for dual staining. We have now added this information to the material and methods section.

The flow cytometry data presented in figures 1D, 4B and 4C show that the majority (90%) of fetal cardiac cells are positive for vimentin. This finding is consistent with the widespread expression of vimentin seen in whole heart sections using IHC. If the gating for vimentin was incorrect, as suggested, and the lower expressing population of cells (67%) actually represents the vimentin negative population, this is would be at odds with the IHC data. We have now added this vimentin IHC data as Supplementary Figure 4 (shown below).

*Supplementary Figure 4. Immunohistochemistry of fetal human heart tissue showing expression of Vimentin at high levels (closed arrowhead) and lower levels (open arrowhead) in the myocardium.*

The aim of figure 4C was to use the colour-coded gating drawn in 4B to highlight the TnI positive cells expressing high and low levels of vimentin. We used a dot plot with TnI on the Y axis and FSC on the X axis to mirror the dot plot in figure 4B from which the gates were drawn, which enable us to show clearly that cardiac cells expressing high levels of vimentin expressed lower levels on TnI, and visa versa. For Figures 4 D-F we wanted to show the dual dot plots with vimentin on the Y axis and the other marker on the X axis as there did not exist two distinct positive populations like vimentin and TnI.

**Figure 5:**

We have now amended the legend for figure 5 to make clear the content of the figure.

Regarding antibody specificity, please refer to our response on page 1. Specifically, the vimentin antibody that was used is very well cited. Furthermore, the isolated primary cardiac fibroblasts stained with vimentin exhibited a staining pattern consistent with intermediate filament proteins. This pattern can be clearly seen in the figure below showing vimentin staining of cardiac fibroblasts during explant migration.

*Vimentin staining of cardiac fibroblasts migrating outwards from an explant of heart tissue. Scale bar: 100 µm*

**Figure 6:**

There are three reasons why we believe that the cells we isolated from cardiac tissue are fibroblasts:

1. The method of explant migration for isolation of fibroblasts is well defined and extensively published. We refer the reviewer to the methods article: ‘An Optimized Protocol for Human Direct Cardiac Reprogramming’ by Garbutt et al 2020 (Cell Press). Here the authors state that by using the method of explant migration they are able to obtain a >90% pure fibroblast population.
2. The morphology of the isolated cells is consistent with a fibroblast morphology showing large, flat, elongated and spindle-shaped cells (see figure 7 and figure above)
3. Whilst vimentin is not specific only to fibroblasts, it is upregulated in these cells. Flow cytometry analysis showed enrichment of vimentin, along with DDR2 and Thy-1, in isolated cardiac fibroblasts, with >96% of cells positive for vimentin.

Regarding the reviewers comment: ‘*Under certain conditions, cardiac fibroblasts can trans-differentiate into cardiomyocytes. It is possible that migratory fibroblasts have begun to trans-differentiate into another cell type. What is the evidence that these are the same cell type as exist in the foetal heart?’*

We accept that any primary cell culture may not fully mimic the cellular phenotype that exists in the heart: however, we have used a well-established, robust technique and at the time of experiment it was difficult to identify a better alternative method that did not solely rely on the use of fibroblast antibodies. Furthermore, the absence of MHC expression in these cells suggests that the fibroblast population does not contain cardiomyocyte cells, either through contamination or transdifferentiation.

**Figure 7:**

We have now added further details of how the isolated fibroblasts were handled prior to immunocytochemistry, PCR and flow cytometry. We have also amended the figure to provide a DAPI co-stain for all the immunohistochemistry images and also changed the figures to higher quality images as requested by reviewer 3.

As suggested by reviewer 1, we have now added a supplementary figure showing dual immunohistochemistry of vimentin and tnI in isolated ventricle fibroblasts (Supplementary Figure 7) (see below):

*Supplementary Figure 7. Dual immunocytochemistry of Vimentin and TnI in cultured primary fetal human ventricular fibroblasts*

Regarding the flow cytometry dot plots for figure 7, as the fibroblasts are enriched for these markers, the fluorescence intensities are subsequently greater and therefore the voltage of the fluorophores will have been adjusted accordingly to ensure all data points are included within the X axis. This is why the levels of the positive peaks differ in figure 7 compared to figure 2. To further support the data in figure 7 and figure 6, we have now added a supplementary figure of dual immunohistochemistry of TnI with MHC in the isolated fibroblasts showing an absence of positive staining of (see below):

*Supplementary Figure 8. Dual immunohistochemistry of TnI and MHC in cultured primary fetal human ventricular fibroblasts*

**Minor Points:**

1. The specific gene/protein names have now been referenced at the beginning of the results chapter for proteins that exists as multiple isoforms.

2. This sentence has now been removed.

3. This typo has now been corrected

**Reviewer 2:**

**1 and 2:** We have now amended the materials and methods section to provide more details for the isolation of fibroblasts, immunohistochemistry, flow cytometry and the antibodies used in the study.

**3:** Vimentin, Thy-1 and DDR2 are all proteins that have been used extensively in previous studies to identify fibroblasts cells. Therefore, we refer to these proteins as ‘fibroblast markers’. Whilst we see enrichment of these proteins in isolated cardiac fibroblasts by IHC, flow cytometry and RT-PCR, their widespread staining throughout the heart suggests these proteins are not fibroblast-specific. We do not believe this is as a result of non-specific antibodies but is concomitant with a more common issue that these so-called ‘fibroblast markers’ are expressed by other cells types. By highlighting this issue in our study, we hope this may encourage others to critically consider if the fibroblast markers investigated in this study, specifically vimentin, should be used to identify and/or isolate cardiac fibroblasts or exclude cardiomyocytes.

To support our IHC data, we have now added a supplementary figure showing the appropriate ‘secondary antibody only’ and isotype controls for IHC (supplementary Fig 2). We agree that co-staining the ‘fibroblast markers’ with GFP from PDGFRα-GFP mice would have been valuable; however, this was not possible for this project.

**4:** We have now improved the quality of the immunofluorescent figures. Due to the large number of added supplementary figures, we feel additional fluorescent IHC images of different regions of the heart would be superfluous for the aim of this study as we believe the flow cytometry data is more informative with regards to identifying the proportions of cardiac cell types.

**5:** Regarding validation of the antibodies, please see our previous response to this on page one. As previously mentioned, the use of reporter mice for antibody validation would have been useful, but was beyond the capacity of this study. We have added more details on how we gated our positive populations by flow cytometry. Figure 2 shows the expression levels of the negative controls (isotype or ‘secondary antibody only’) relative to the positive populations.

**6:** There are a number of alternative markers for fibroblasts that could have been investigated, including mEF-SK4, PDGFRα and Col1a1. We chose vimentin as it is one of the most common markers used to identify cardiac fibroblasts (Ieda et al 2009 and 2010, DOI: doi.org/10.1016/j.devcel.2008.12.007 and 10.1016/j.cell.2010.07.002). We then chose to focus on proteins that had previously been identified as specific to cardiac fibroblasts (rather than fibroblasts in general): **Thy-1**: Hudon-David et al., 2007 DOI:10.1016/j.yjmcc.2007.02.009; **DDR2**: Banerjee et al., 2007. DOI: [10.1152/ajpheart.00514.2007](https://doi.org/10.1152/ajpheart.00514.2007)).

**7:** We chose to categorise the TnI^Low^/MHC^-^ population of cells as the ‘non-myocyte population’. Due to the lack of a fibroblast-specific marker and the fact that we only focused on the main cells types of the heart and have not looked at other cell types, including mural cells and immune cells, we could not speculate the cellular composition of this population, other that it is absent of cardiomyocytes. Based on the fact that this population expressed TnI at low levels (and we know that the TnI^Low^ population expressed vimentin at high levels), we can propose that this population consists of endothelial cells and fibroblasts/smooth muscle cells. However, further investigations are required to determine this. We have now added a section at the end of the discussion to discuss this and other limitations of the study.

**8:** We agree that the expression of α-SMA in the cultured fibroblasts may be due to the culturing condition of these cells and have now added this to the discussion. In the absence of a reliable fibroblast marker that is expressed on the surface of fibroblasts to facilitate sorting of these cells for downstream applications, the extraction of fibroblasts from human heart tissue via *in vitro* explant migration is currently the only viable option.

**9: -*Please tone down your conclusions and use non-speculative language as possible in your discussion, for example, "suggesting a role of troponins in non-muscle cells distinct from that of cardiomyocytes and skeletal muscle cells", "vimentin expression may be a property of cardioymyocytes of the developing heart only.".***

We have now changed the sentence discussing cTnI to read:

*‘We observed a non-striated pattern of expression of cTnI in cardiac fibroblasts suggesting a lack of sarcomeric organisation, however, it remains unclear how this may alter the role of cTnI in these cells.’*

We have now changed the sentence discussing vimentin to read:

*‘However, it remains unclear why vimentin expression has only been observed in fetal and not adult cardiomyocytes.’*

**10:** We argue that the positive expression of MHC in 75% of cardiac cells from a total of 21 hearts is reasonable evidence supporting the estimate that 75-80% of cardiac cells of the early fetal human heart are cardiomyocytes. Furthermore, the expression of high levels of TnI occurred in 79% of cardiac cells, from a total of 14 hearts. The antibodies used to provide these data produced a sarcomeric staining pattern in whole heart sections by IHC and have been extensively referenced by previous studies (11 times for MHC and 110 times for TnI). Pinto et al., (2016) estimated the proportion of cardiomyocytes to be approximately 54% based on IHC staining alone, and no flow cytometry analysis. We appreciate that 75-80% is significantly higher than previous estimations, but we remind the reviewers that we are comparing our findings to data carried out on adult human heart, and the cellular composition of the early developing heart is likely to be different from adult. The only other study to investigate cellular composition in the developing heart was by [Suryawanshi](javascript:;) et al (2020) who used only n=3 foetuses of much later gestational age (19-22 week of gestation).

As mentioned previously, we limited our investigations to the four main cells types of the heart and therefore we cannot comment of the cellular contribution by other cell types. We now discuss this in the manuscript.

**11:** We have now corrected this typo.

**Reviewer 3:**

**Major Comments:**

**Specific Concerns:**

***p2: Abstract***

***p2, line 26: …which remains largely unknown: here you should include something like “at least in humans”***

We state: ‘Reliable identification of fetal cardiac cell types using protein markers is important for understanding cardiac development and delineating the cellular composition of the **human heart** during early development, which remains largely unknown.’

***p2, line 30: …Myosin Heavy Chain: you should specify if you mean the alpha or beta isoform***

This has now been amended

***p2, line 30/31: you should use a consistent nomenclature to describe gene or protein names; (e.g. sometimes you only use the abbreviation, sometimes the complete name is written…).***

This has now been amended. When referring to the specific protein, we use the appropriate abbreviation after the first incidence of the full name and the abbreviation has been introduced.

***p3: Introduction***

***p3, line 63: you should shortly discuss the different embryonic origins of fibroblasts which contribute to fibroblast heterogeneity***

We have now added: ‘*The phenotypic plasticity of cardiac fibroblasts depends on their developmental origin (epicardium, endocardium or neural crest) as well as their location within the myocardium’*, referencing Moore-Morris *et al* (2016). Origins of cardiac fibroblasts. *Journal of molecular and cellular cardiology* 91, 1-5, doi:10.1016/j.yjmcc.2015.12.031 (2016).

***p4, line 72: DDR2 is also a surface Protein and is mainly found on the plasma membrane (by the way: this sentence should also be shortened, maybe build 2 sentences.)***

We have amended this section to make it more clear and concise.

***p4, line 84: CD31 is not specific for endothelial cells (is also expressed in macrophages), alphaSMA is not specific for Smooth muscle cells but also expressed in activated fibroblasts. Please relativize that.***

We have now added: *‘However, CD31 is also present on macrophages, and α-SMA is upregulated in activated fibroblasts, termed ‘myofibroblasts’, that respond to injured cardiac tissue.’*

***p4, line 93: estimate not “estimare”***

This has been corrected

***p5: Materials and Methods section****:*

***the whole section should include more experimental details so that one is able to follow the experiments
a table with the specimen used should be included***

We have now added more details to this section, including a new antibody table with the corresponding controls (Supplementary table 1). We have also produced a new table displaying all the FACS data percentages, the tissue ages used and the individual flow cytometry percentages of each sample (Supplementary Table 3)

***p5, line 106: 70µm strainer: this is a size discrimination, did you check if you lose cardiomyocytes in this step? Or are fetal cardiomyocytes smaller than 70µm?***

Initial filtering after tissue homogenisation with a 70µm strainer ensured no clumps in the cell suspension. Additional filtering through 35 µm filter is essential prior to reading them by flow cytometry so as not to obstruct the FACS nozzle with debris. Cardiomyocyte width is estimated at 20-35 µm (*in vivo*, not in cell suspension) and therefore should easily pass through this filter. As we saw a significant number of cells positive for MHC, we do not believe this procedure led to a loss of cardiomyocytes from the samples.

***p5, line 109: heading should be “Fibroblast Isolation and Cell Culture”***

This has now been amended (on page 7, line 152)

***p5, line 115: you should please note centrifugation speed in [g] not in rpm, since rpm is dependent on the rotor radius***

This has now been amended

***p5, line 117: 35µm strainer: this is a size discrimination (even more than mentioned above line 106), did you check if you lose cardiomyocytes in this step? Or are fetal cardiomyocytes smaller than 35µm?***

Please see response above

***p5, line 133: as already noted above: how did you check specificity of antibodies? E.g. positive controls? Double stainings?***

Please refer to our response regarding antibody specificity and validation on page 1 of this rebuttal.

***p5, line 135: as already noted in the abstract: is it alpha or beta MHC or does the antibody detect both isoforms?***

Please see our response below to ‘p7, line 159’

***p7, line 143: please note in that paragraph that results of the RT-PCR were visualized on an XXX% agarose gel and the fluorescent dye you used….***

This has now been added to the end of the RT-PCR section

***p7: Results***

***p7, line 157: TnT is not shown in Fig1A? Missing? Not stained?***

We used cTnT to validate the flow cytometry data of cTnI, showing high and low positive populations (Supplementary Figure 3). We did not do any additional IHC with this antibody, although this antibody is well characterised, having been cited in over 71 publications.

***p7, line 159: now you write that you stained alphaMHC. There are two isoforms of MHC: alpha and beta. The beta isoform is more relevant in fetal cardiac tissue while the alpha isoform is more typical for adult cardiac tissue. Could you please discriminate between the two isoforms? Are there antibodies that are specific for each isoform? Would be interesting to analyze the amount of beta versus alpha…***

This is an interesting question, however, determining differences between alpha and beta MHC distribution was not part of this study. The antibody used to detect myosin heavy chain is the widely used monoclonal antibody MF20 (please see antibody table). MF20 is a pan myosin heavy chain antibody that labels the myocardium and has been cited by many others as a cardiomyocyte marker (Please see page 2 of this rebuttal). We appreciate you asking for clarity on this issue; we have now ensured that all references to this protein in the text state ‘MHC’ and not ‘α-MHC’.

***p7, line 161: since Thy1 is a surface protein, it should not be localized in the cytoplasm…. Vimentin should mark fibres of the cytoskeleton (However, I cannot recognize these patterns since the picture quality is so poor). In addition, there is DDR2 in the figure (not mentioned in the text).***

We have now amended this figure to include images of higher quality that better represent the expression we saw in the heart. We agree that as a surface marker we would expect to see Thy-1 on the cell surface, however, we have identified other published IHC and immunocytochemistry that show Thy-1 expression not localised only to the cell surface (Sidney et al., 2015, DOI: https://doi.org/10.1016/j.jcyt.2015.08.003.; Pietro et al., 2020 DOI: https://doi.org/10.3390/ijms21124356). This suggests possible internalisation of the receptor where it may play an alternative role. We must also consider that the thickness and cellular density of the tissue, which will result in sectioning of cells along different planes and therefore surface staining can be interpreted as cytoplasmic based on localisation relative to the nucleus.

We have now also added in commentary of DDR2 expression pattern in the text.

***p7, line 162: how do you know that these antibodies stain endothelial cells or smooth muscle cells? This is not the thing you can read from this picture. You only see that the antibodies stain cells from vessel walls.***

Dual staining of α-SMA and CD31 showed that they stain distinct areas of the vessels (Please see new Supplementary Figure 6). CD31 stains the inner lining and α-SMA stains the outer lining of the vessel, corresponding to the expected endothelial cells and smooth muscle cell layers.

***p8, line 181/182 (legend for Fig 2I): Percentages of MHC and TnI are not the same as mentioned in the text???? Please correct.***

We have checked that the percentage of MHC and TnI in the text are the same as those displayed in the figure.

***p8, line 187/188: Please indicate percentages for TnT high and low expressing populations. By the way, the same holds true for MHC. Can you also add this into the Suppl Figure?***

Unfortunately, we are not able to source this data.

***p8, line 188ff: You should do stainings for fetal aorta/pulmonary artery not only with TnI antibodies but also with Vimentin, TnT and MHC.***

For the purpose of this manuscript we wanted additional evidence that the population expressing TnI at lower levels was a non-myocyte population and therefore we did TnI staining in tissue absent of cardiomyocytes. We agree there is potential to do additional staining of the aorta/pulmonary artery with other markers, but we believe this is beyond the scope of this manuscript that is focused on cell populations of the heart and not the outflow vessels.

***p9, line 213 (corresponding to Fig 4 C): Could you please include gates into this dot plot similar to the other dot plots in the same Figure? For Fig 4D: in the dotplot itself there should be aSMA/Vim not TnI/Vim, right?***

We have now added a gate to Fig 4C distinguishing the TnI^+^ and TnI^-^ populations. We have also amended the label of Fig 4D.

***p9, line 221: I know that you regard Vimention as a fibroblast marker. However, this marks cells from mesenchymal origin. Thus for a proof of principle you should also double stain Vimentin and DDR2 and Vimentin and Thy1.***

We do not regard vimentin to be a specific fibroblasts marker; however, as vimentin is regularly used as such, we chose to investigate its expression in the fetal heart and our data offers further evidence that vimentin is expressed in other cells types other than fibroblasts.

***p10, line 234: However, Vimentin intensity is not different in Fig5A (in the FACS you showed Vimlow for TnI high cells.***

We agree that based on the flow cytometry data we would expect to see the low and high populations mirrored by IHC on heart tissue, as we see with TnI. It is possible that despite lower expression of vimentin in the myocardium, the density of the cells in the compact myocardium is such that the fluorescence intensity appears similar to the blood vessels.

***p11, line 255: Fetal heart tissue also expresses THY1 and DDR2; the gene name for aSMA is ACTA2 (in Fig 6 you should not use the protein names but the gene names which are sometimes different). Human gene names are written in capitals (e.g. THY1 not Thy1)***

We have now amended the nomenclature in the text and the figure

***In Fig 6 you should show a negative control and an amplification control such as GAPDH or BetaActin for all the lines so that one can decide if the differences in the band intensities are really reflecting differences in gene expression and not only differences in amplification efficiency, etc.***

Housekeeping controls were used for reverse transcription PCR (RT-PCR), however, unfortunately the primary data (gel images) are no longer able to be accessed. We used RT-PCR to show the presence or absence of transcripts and to confirm there was no contamination of CMs in the fibroblast population. We have now amended the text to ensure we only discuss the RT-PCR data in a qualitative manner.

***p12, line 267: you should write “….in fetal cultured ventricular fibroblasts”***

Amended

***p12, line 268: THY1 and DDR2 are missing***

Amended

***p12, line 272: you should also stain for CD31 and MHC and check if cultured fibroblasts are negative for those antibodies (important for specificity of those antibodies).***

We have now added a new supplementary figure showing dual staining of cultured fibroblasts with TnI and MHC (showing no staining of MHC) (Supplementary Fig 8). Please see page 8 of this rebuttal.

***Figures
The overall quality of pictures in the figures is not very good, especially the immunohistochemical stainings are rather blurry and overexposed. Please improve the quality of these images (Fig 1, Fig 5, Fig 7, Suppl Fig 2), e.g. by taking pictures with a confocal microscope.***

We have now improved the quality of the images, which was due to an issue with exporting them as TIFs.

***The Figure Legends should also be improved. In the legends you should shortly describe what you see in the pictures, so that you can understand without reading the text.***

We have amended the figure legends where necessary. We believe these legends are consistent with those from previous PlosOne publications. If the editor would like us to amend further we are happy to do so.

***p12 Discussion***

***p13, line 285: you cannot say that cTnI lower levels were confirmed in non-myocytes by RT-PCR, since you have no housekeeping gene in your RT PCR as a normalization control.***

This has now been amended.

***p14, line 315: why should there be myofibroblasts in a fetal heart? This is a cell type that is usual representing some kind of pathology in a heart…***

This has now been amended.

***In the discussion you should compare your results from human fetal hearts to results from murine fetal hearts and of course to state-of-the-art single cell studies on the cellular composition and gene expression of human fetal hearts.***

This has now been included.
