# Supplementary material for: Defining cardiac cell populations and relative cellular composition of the early fetal human heart
Source: PLoS One. 2022 Nov 30;17(11):e0259477. doi: 10.1371/journal.pone.0259477 (PMC9710754; doi:10.1371/journal.pone.0259477)
Supplement: Attachment — Submitted filename: Response to Reviewers #2, Dewing et al July 2022.docx [file pone.0259477.s013.docx]

**Response to Reviewers**

***Reviewer #1:*** The authors have added further details to the methods section and provided a little more clarity in some figure legends. They have also inserted some additional supplemental data, including negative controls for IHC staining and a table showing the antibodies used. Together these are useful to clarify what was done. In particular, they have explained what the negative control data for the flow analysis represents. This is helpful, strengthens their conclusions and addresses some of my original concerns. I believe this revised version now satisfies PLOS guidelines on minimal reporting.

However, they have made no changes to the interpretation of the data based on the feedback received. The figures, abstract and main text remain largely unchanged. Despite providing a detailed rebuttal to the reviewers, very few changes have been made to the manuscript itself. Most of the points made in the rebuttal are omitted from the main manuscript. For this reason, some of my original concerns remain outstanding.

In general, I feel that the manuscript that the manuscript would benefit from a re-write. It remains thin on experimental details and I feel that some sections could be explained more clearly. The sub-headings could be changed to describe the key findings rather than the methods used. More explanation is needed in the text of the fibroblast explant culture method and also of the rationale of analysing different parts of the heart.

**Authors’ response:**

We appreciate the reviewer taking the time to submit a detailed review of our manuscript and we are disappointed that they feel we have not made sufficient changes to the manuscript. Based on the reviewer’s first round of comments we had made the following amendments:

- Updated the methods to provide additional detail
- Provided a negative control supplementary figure for IHC data
- Provided a complete antibody table
- Provided a complete list of all flow cytometry data
- Added a IHC figure showing distinct staining of CD31 and a-SMA
- Added a FCS x SCS flow cytometry dot plot supplementary figure
- Added a IHC figure showing high and low expression
- Added a ICC figure showing dual staining of vimentin and TnI in fibroblasts
- Added a ICC figure showing dual staining of MHC and TnI in fibroblasts

Furthermore, in the original rebuttal we addressed the reviewers concerns by written reply regarding the following issues, trying to provide an explanation that would justify why on balance a change to the manuscript was unnecessary:

- Antibody validation and specificity
- The interpretation of the flow cytometry data for figure 2
- The differences in fluorescence intensity of different markers
- The differences in appearance of flow cytometry peaks between markers
- That gating of MHC and CD31
- The shape of the gates
- The interpretation of figure 3
- The expression of TnI in non-myocyte tissue, citing other articles with the sae findings
- Justification of dual vimentin populations using supporting IHC data
- Explanation of the data presentation of figure 4
- Expression of vimentin in fibroblasts consistent with intermediate filament staining pattern
- Justification of explant migration for fibroblast isolation
- Fluorescence intensity of fibroblast flow cytometry in figure 7

The reviewer has again raised concerns relating to the level of detail provided in the flow cytometry gating, and also our interpretation of the data, and offers an alternative interpretation. We do agree that explanation of how the raw data is gated is critical and we have now provided an additional supplementary figure that shows how gating was achieved using flow cytometry dot plot analyses. This is described in more detail under the section for figure 2 below, which clarifies the gating strategy.

It has been helpful to be challenged over our interpretation of the data relating to the flow cytometry peaks observed for cardiac troponin I and whether some peaks represent populations of negatively stained cells, which would of course require a re-evaluation of our conclusions and re-write as suggested, if the alternative interpretation was correct.

In this regard, as suggested by reviewer 1, adding the data for unstained cells is useful in understanding the baseline autofluorescence of the total cardiac population, which lies between -10^2^ and 10^2^ on the X axis, which is in a similar range as the negative controls used for gating. We are including the unstained cell data, detected at three separate laser wavelengths, which correspond to the fluorophores APC, FITC and PE, and have added this data to supplementary figure 1, but resisted showing them as a composite in figure 2 in order to prevent a complexity of the graphic. Given that we are presenting the unstained data, we hope this is acceptable.

Regarding the interpretation of the cTnI data, we wish to explain why we believe there is a peak representing high cTnI expressing population and a peak that represents low cTnI expression (in addition to negative control). Examination of the cells from the aorta/pulmonary artery (AO/PA) shows two distinct peaks: a smaller peak at the same fluorescence intensity as the negative control (-10^2^ and 10^2^), and a second, larger peak with a fluorescence intensity of 10^3^. The fluorescence level of the second AO/PA peak is 1000 times greater than the first (control) peak. Were this presented as a “standalone” experiment, the result would be interpreted as showing a distinct peak with characteristics of a sizeable population of cTnI positive cells. This is consistent with the immunohistochemistry data of cTnI positive staining in the wall of the aorta (Fig 5A). Examination of the flow cytometry cTnI staining of the heart (atria and ventricle) shows three peaks. The smallest peak is at the same fluorescence intensity as the negative control peak (and unstained cells in supplementary figure 1 as discussed above). A middle peak, that is distinct and significantly higher than the negative control but importantly of similar level to the positive AO/PA cells. In fact it has a higher level of staining than the AO/PA cells. And a third peak, representing a cell population with the highest level of staining. The reviewers suggests that the cTnI middle peak represents a cell population with negative cTnI staining; if this was the case we would not expect the positive AO/PA population at a similar intensity. The reviewer’s alternative interpretation could also lead to a conclusion that the AO/PA peak is negative staining and there are no positively stained cells, which we believe to be incorrect. Our interpretation is supported by the IHC data in Fig 5A, as well as our RT-PCR data in figure 6A, which shows expression of troponin genes in the absence of α-MHC transcript in ventricular fibroblasts: this confirms the presence of cTnI and cTnT in non-myocyte cells that are uncontaminated by cardiomyocytes.

The first set of comments from reviewer 1 includes the statement “*Troponin is used only by striated muscle and not by smooth muscle*” and we wonder whether this forms the basis for the alternative interpretation and questioning of our conclusions. As mentioned in the revised discussion, several recent studies have identified troponins in non-myocyte cells: single cell transcriptomics has identified TNNT3 to be specifically expressed in endothelial cells of the fetal heart, which was confirmed by IHC (Suryawanshi et al, 2020), and cTnT immunostaining of cardiac fibroblasts was observed in 17 gestational week fetal tissue (Cui et al 2019). This is independent data that troponins are expressed in non-myocyte cells in the fetal heart and further supports our interpretation of the data.

With regards to experimental detail provided, we have acted on the reviewers’ comments and have added text to the revised manuscript, which can be seen in the uploaded tracked changed version. In particular we hope the level of detail describing the method used to isolate migrating cells from cardiac tissue provides sufficient detail. The details of the flow cytometry procedure are significantly more detailed than most articles we could find.

Regarding the reviewer’s comment: ‘*More explanation is needed in the text of … the rationale of analysing different parts of the heart.’* We appreciate this comment and have therefore been more explicit and define which parts of the heart were included in experiments. To clarify, when dissecting the cardiac tissue obtained from the HDBR in Newcastle, the aorta and pulmonary arteries were dissected away from the ventricles and we continued our analyses on the atrial and ventricular tissue (referred to as heart tissue in the manuscript), as described in the methods. The rationale of using aorta and pulmonary artery tissue separately for flow cytometry and IHC was to investigate cells uncontaminated by myocardial content enabling reference to data showing troponin I expression in non-myocytes. We have now added the following sentence to the methods:

***‘The use of aorta and pulmonary artery tissue separately for flow cytometry enabled the investigation of cells uncontaminated by myocardial content.’***

With regard to the comment “The sub-headings could be changed to describe the key findings rather than the methods used.” We acknowledge that some articles choose to have subheadings that summarise the key finding of that section, however, after consideration we felt that this was not appropriate for this style of manuscript owing to the fact that each section describes several different findings with the technique used being the common theme. Therefore, such a style of subheading would not fully capture the findings. Furthermore, we found that Pinto *et al.,* also used similar subheadings for their study investigating cellular composition of the mouse and human heart, that included ‘Immunohistochemical Analysis of Cardiac Cellular Composition’ and ‘Flow Cytometry Analysis of Nucleated Metabolically Active Cells’. However, we are happy to amend the subheadings should the editor feel this is necessary for PloS articles.

**Detailed Comments**

**Fig1**

Negative controls for these antibodies are now shown in Fig S2 and look fine. This figure should be referred to in the text.

Supplementary Figure 2 (now supplementary figure 3) was referred to in the methods section, under ‘immunohistochemistry’, however we have now also referred to this figure in the results section.

**Fig2**

This figure has not been modified in the revised version.

The authors have now told us what the negative control is and this has answered some of my concerns. However, the positioning of the gate remains arbitrary and therefore all of the concerns I raised in my original review regarding this remain outstanding.

In their rebuttal, the authors refer to a figure from Banerjee 2007, which they state was used to design their own figure. I would therefore like to point out that in Banerjee the position of the gate is not arbitrary, but is aligned with the end of the unstained control cells, and is in the same position in every panel. The position of the gate in this current manuscript is arbitrary. (the authors correctly point out that Banerjee does not use an isotype/secondary control to position the gate. Their method is indeed an improvement, but only if information from this control is used to inform positioning of the gate).

We have sought to clarify the gating strategy, which we explain below and in addition explain the modifications since our initial rebuttal: under ‘Fig.3, 3a-e' we stated:

***‘The gating for all flow cytometry data in this manuscript was drawn based on the dot plots, not the histograms, as this gives a more accurate representation of the distribution pattern of the negative cell populations. We have now added this information into the methods section.’***

The ‘gates’ in figure 2, with the overlying percentages do not represent the actual gating used to define the cell populations, as all gating was performed using dot plot graphs, which is more accurate. This is due to the variation in cell size (FSC) of the heterogeneous cardiac cell population which means setting a straight rectangular gate at a specific fluorescence intensity on a histogram does not fully capture the distribution of positive or negative cells. Rather than gates being arbitrarily defined, their position is defined by the population of cells in the negative controls (isotype or secondary only). As shown in the new supplementary figure 2 (below), the position and shape of the gate depends on negative population. It is because Banerjee et al only use unstained cells as a control that a “constant gate” is possible and presented in their figure. The ‘gates’ shown in the histograms of Figure 2 are therefore used to approximate these positive and negative populations; however, the percentages displayed in figure 2 are the actual percentages measured from the dot plot analyses. This information had been added to the methods. We have now also added the following sentence to the figure 2 legend: **‘*The histogram gates are representative of the positive and negative cell gating that was determined using flow cytometry dot plots.’***

We appreciate you highlighting that this was not clear in the manuscript and we have now changed all the ‘gates’ on the histogram to start where the negative control ends to prevent confusion, which reflects the gating strategy. We have also now added another supplementary figure that shows the gating of the cell populations using the dot plots as described (supplementary figure 2) (see below). We believe the recommended changes provide clearer detail of the gating strategy.

*Supplementary figure 2*

Much of the paper concerns the existence of two populations of cells (high and low expression of a given marker), therefore the decision to arbitrarily exclude cells showing low MHC expression is bizarre.

The positioning of the gate in other panels also appears rather random, sometimes overlapping the tail of the negative peak, sometimes not.

We apologise for the confusion regarding the gating of MHC and we thank the reviewer for highlighting this issue. Despite the original figure highlighting only the far peak as MHC^+^, the actual total percentage of positive MHC cells (75%, n= 21) represents all cells that were positive above the negative control, as shown in the gating strategy for the new supplementary Figure 2. Initially, we chose to be more specific for MHC because of the narrow far peak and very low and broader first peak. Unlike TnI, which has an obvious distinct population of cells at a lower fluorescence intensity, there is not a distinct cluster expressing lower levels of MHC, and as the fluorescence intensity of the larger population is very high (>10^4^), it is unclear whether the cells with a lower level of expression constitute a distinct population and therefore grouped MHC positive cells. However, we accept the reviewer’s comments and we cannot exclude the possibility that the first smaller peak may represent a very small population of positive cells expressing MHC at lower levels. We did not feel our data justified claiming the existence of what would be a very small population and therefore make no comment in the manuscript. We hope the positioning of the gate on the revised figure 2, and the additional supplementary figure makes it clearer to readers how MHC positive populations were selected.

Fig2a: The purpose of this figure is to show autofluorescence of unlabelled cells. However, the x axis is unlabelled so it is unclear which channel this corresponds to, making this panel meaningless. The authors used antibodies with a variety of different fluorophores, therefore, we need to see autofluorescence data plotted for each channel. It would be better to plot these unlabelled cells on each panel along with experimental and control antibodies.

As mentioned on page 1 of this rebuttal, we have now modified figure 2 to remove the representative unstained histogram and instead added three separate histograms of unstained heart samples (with each fluorophore shown on the X axis) to supplementary figure 1. As we do not use the unstained samples to inform cell gating, we do not feel including the unstained control on each histogram is useful and may confuse the interpretation of the data. We have now also added the fluorophore to the X axes of the histograms in figure 2. Please see below the revised figures as described above:

*Revised Figure 2*

*Revised Supplementary Figure 1*

**Fig3**
No Fig 3 is provided.

This has now been uploaded

**Fig 4**

OK

**Fig 5**

It is still not too clear what we are looking at. It would be better if the panels were at the same mag and showed adjacent sections of the same tissue. One concern I have here is that in Fig 5a the line dividing the myocardium from the blood vessel appears to be based on differing expression levels of cTnI, yet this difference is then given as evidence that non-cardiomyocytes express cTnI. This is circular logic, and could be avoided if another image showing MHC/SMA were included and used to draw the line.

We did not define the border using the differential expression of TnI. The line dividing the myocardium from the AO/PA is defined by the tissue structure and density of cell nuclei, which can be seen with DAPI staining as well as vimentin staining. Please see figure below that is an additional example, which shows how a dividing line may be drawn based on the nuclei distribution of DAPI and the staining pattern of vimentin, and does not include TnI. We agree that a further experiment that stained MHC or α-SMA to an adjacent serial section would have been helpful but unfortunately this is not possible. We appreciate why the reviewer has raised this concern, that circular argument of TnI expression has being used, but this is not the case: the line of demarcation is created without reference to TnI expression.

**Figs 6, 7**

Some of the expression patterns shown in Fig 7 are quite odd. cTnI is shown expressed in the nuclei of these fibroblasts – this is very odd for a structural protein and not the expected pattern. The expression pattern shown in Fig S7 is quite different to that in Fig 7. However, neither of these are points are mentioned in the text at all. It looks like something has gone wrong here.

I think Figs 6 and 7 could be combined into one, perhaps with a diagram to explain the methods. Co-expression data shown in Fig S7 is key, so could be moved to main figure.

Thank you for pointing out this issue in figure 7. You are correct that the wrong image was shown for cTnI and we apologise for this oversight. We have now changed the image for cTnI expression in ventricular fibroblasts to the correct image. We have also combined figure 6 and 7 as suggested and also added in supplementary figures 7 and 8 to create a larger figure that is more informative. Please see below:

***Reviewer #2:*** I would like to thank the authors for addressing all of my comments. I have no additional suggestions.

***Reviewer #3:*** The manuscript “Defining Cardiac Cell Populations and Relative Cellular Composition of the Early Fetal Human Heart” tries to analyze commonly used markers for the main cardiac cell types, such as cardiomyocytes, cardiac fibroblasts, endothelial cells and smooth muscle cells, in human fetal hearts (< 12 pcw ). The findings are mainly based on antibodies used for immunohistochemistry as well as flow cytometry analyses.
In the revised version of the manuscript, the authors improved the picture quality for immunohistochemistry. The authors also added more details to the Material & Method’s section. In addition, the authors added a paragraph to the discussion where they compare their findings about the human fetal heart to mouse data and to state-of-the-art single cell data.
However, there are still several concerns from my side. Unfortunately, the authors did not address some of my major comments. They did not even answer to points such as “In addition, it would be helpful to directly compare the findings to adult specimen. In the discussion, the authors talk about the differences in cell composition and marker expression between fetal hearts and adult hearts. But given the different antibodies used for detection of cell populations and different methods used for dissociation of tissues, such numbers might be highly variable. Please perform immunohistochemical stainings and flow cytometry analyses with the same antibodies on adult specimen.”

The comment “The newest reference cited by the authors is from the year 2019. However, science is rapidly progressing, especially on the topic of cardiac development and fetal hearts since the heart is one of the most important organs and the one developing first in embryogenesis. References should be updated and newest publications, especially concerning single cell analysis of fetal human hearts should be included“ was addressed by adding one more reference (from 2020). However, also here the authors did not answer to my comment.

I see that the authors might feel annoyed if they get lots of comments from the reviewers. However, as a reviewer, one invests time to perform this review and really tries to offer comments that help to improve the manuscript.
At least, the authors addressed some of my specific concerns. However, sometimes they claim that they have changed a certain paragraph or statement but they did not.
In addition, I see it as a significant problem that, in several cases, the authors are not able to present the source or original data or images.Please find the problematic points in the following.

**Authors’ response:**

We apologise for not addressing all of your concerns. With regards to your original comment about the flow cytometry gating strategy, we have now added a new supplementary figure (sup fig 2) that presents the flow cytometry dot plots for the cell markers investigated, alongside their corresponding controls and the gates used to determine the percentage of positive cells.

With regards to the comment ‘*it would be helpful to directly compare the findings to adult specimen’…* *Please perform immunohistochemical stainings and flow cytometry analyses with the same antibodies on adult specimen.’*

The reviewer indicates they had previously commented that comparison with adult human tissue would have value. We had not been able to act on this comment and unfortunately did not provide an explanation in the first rebuttal which was an omission on our behalf. Taking this comment as a general point, we of course agree that it would be helpful for a study to investigate both fetal and adult tissues, but this assumes practical aspects of tissue accessibility. We agree that comparison with adult heart tissue would be of value, but as a requirement for publication it is not practical. The expectation that a new study could be created (necessary to handle tissue rapidly and develop adult tissue/cell fixation and disaggregation protocols, with ethical approval, appropriately funded, accessing normal human adult hearts with informed consent within the time frame set by PLoS for a response to reviewers’ comments) is surprising and we suggest unreasonable if it is to be set as a requirement for publication. The aim of this study was to focus on the cardiac populations of the developing heart; we defend our justification of antibody specificity and believe our findings are robust, offering a valuable contribution to this field. We have now added more details in the discussion about the most recent studies that have investigated adult cardiac populations and the techniques used.

The comment “The newest reference cited by the authors is from the year 2019. However, science is rapidly progressing, especially on the topic of cardiac development and fetal hearts since the heart is one of the most important organs and the one developing first in embryogenesis. References should be updated and newest publications, especially concerning single cell analysis of fetal human hearts should be included” was addressed by adding one more reference (from 2020). However, also here the authors did not answer to my comment.

Regarding the references, we agree that it is important to ensure that the references reflect the evolving landscape of the area of research. In addition to the added reference of Suryawanshi et al (2020), we have also added an article by Tucker et al (2020) that used RNA seq to investigate adult cardiac populations, which we now mention in the discussion. We have struggled to find more recent studies using single cell analysis to investigate cardiac populations of fetal and normal adult human hearts, in contrast to studies investigating disease states. We have however added a 2021 review article by Marín-Sedeño *et al* that discusses using single cell transcriptomics to understand the adult mammalian heart.

**Specific Concerns:**

my comment: p4, line 84: CD31 is not specific for endothelial cells (is also expressed in macrophages), alphaSMA is not specific for Smooth muscle cells but also expressed in activated fibroblasts. Please relativize that.

authors: We have now added: ‘However, CD31 is also present on macrophages, and α-SMA is upregulated in activated fibroblasts, termed ‘myofibroblasts’, that respond to injured cardiac tissue.’
- Unfortunately I cannot find that in the text. Might the authors have sent a wrong version of their revised text?

We apologise for this. It is unclear why these comments were not present in the revised manuscript, when some of them were. We have now added this sentence to the text.

p5: Materials and Methods section:

my comment: p5, line 109: heading should be “Fibroblast Isolation and Cell Culture”
authors: This has now been amended (on page 7, line 152)

-it has not been changed. Again: wrong text version?

Apologies. We can now confirm this sentence is added to the text.

my comment: p7, line 143: please note in that paragraph that results of the RT-PCR were visualized on an XXX% agarose gel and the fluorescent dye you used….

authors: This has now been added to the end of the RT-PCR section

- it has not been added. Wrong text version?

Apologies. We can now confirm this sentence is added to the text.

my comment: p7: Results

p7, line 157: TnT is not shown in Fig1A? Missing? Not stained?

authors: We used cTnT to validate the flow cytometry data of cTnI, showing high and low positive populations (Supplementary Figure 3). We did not do any additional IHC with this antibody, although this antibody is well characterised, having been cited in over 71 publications.
- Then it should not be mentioned when talking about the IHC results in Figure 1. Please change that.

Apologies for this oversight. We can now confirm this has been removed from the beginning of this results section.

my comment: p7, line 161: since Thy1 is a surface protein, it should not be localized in the cytoplasm…. Vimentin should mark fibres of the cytoskeleton (However, I cannot recognize these patterns since the picture quality is so poor). In addition, there is DDR2 in the figure (not mentioned in the text).

authors: We have now amended this figure to include images of higher quality that better represent the expression we saw in the heart. We agree that as a surface marker we would expect to see Thy-1 on the cell surface, however, we have identified other published IHC and immunocytochemistry that show Thy-1 expression not localised only to the cell surface (Sidney et al., 2015, DOI: <https://doi.org/10.1016/j.jcyt.2015.08.003>.; Pietro et al., 2020 DOI:https://doi.org/10.3390/ijms21124356). This suggests possible internalisation of the receptor where it may play an alternative role. We must also consider that the thickness and cellular density of the tissue, which will result insectioning of cells along different planes and therefore surface staining can be interpreted as cytoplasmic based on localisation relative to the nucleus.

- I agree with the author’s remarks. However, the text says Thy1 is “predominantly” found in cytoplasm (I would not agree with that from what I see on the pictures, now that the picture quality is improved). You might write: is “also” found in cytoplasm….
authors: We have now also added in commentary of DDR2 expression pattern in the text.
- No, I cannot find that.

We have now added the following sentence to describe thy-1 staining in figure 1:

***‘The fibroblast markers showed extensive staining throughout the heart tissue, with vimentin localised to the cytoplasm and the membrane protein Thy-1 also showing cytoplasmic staining (Fig 1B).’***

We apologise for the commentary of DDR2 not being in the submitted manuscript. It appears these issues were due to a merging error between separate versions of the manuscript. We can now confirm that we have added the following sentence:

***DDR2 expression was seen throughout the myocardium and was localised to the nucleus and the cytoplasm.***

my comment: p8, line 181/182 (legend for Fig 2I): Percentages of MHC and TnI are not the same as mentioned in the text???? Please correct.
authors: We have checked that the percentage of MHC and TnI in the text are the same as those displayed in the figure.
- But the bar graph in Fig 2I shows not the correct percentages for MHC (the text says 75%, the bar graph says 65%) and TnI.(the text says 93%, the bar graph says something less than 40%). Please correct that.

Thank you for clarifying this concern. We have now corrected the data in the bar graph for figure 2.

my comment: p8, line 187/188: Please indicate percentages for TnT high and low expressing populations. By the way, the same holds true for MHC. Can you also add this into the Suppl Figure?

authors: Unfortunately, we are not able to source this data.

-then the data should not be used for a manuscript.

We performed flow cytometry on TnT to demonstrate the existence of two distinct positive populations similar to that for TnI. Initially we were unable to source the raw .fcs files for TnT but we have now managed to find n=2 .fcs files that we have analysed to determine the percentages of the high and low TnT positive populations. Please see below the histograms and dot plots for these two samples. The average percentage for the TnT Low population was 28% and the average TnT High population was 64%. We have now added these averages to supplementary figure 3 (see below, right) and added this information to the manuscript text.

my comment: In Fig 6 you should show a negative control and an amplification control such as GAPDH or BetaActin for all the lines so that one can decide if the differences in the band intensities are really reflecting differences in gene expression and not only differences in amplification efficiency, etc.

authors: Housekeeping controls were used for reverse transcription PCR (RT-PCR), however, unfortunately the primary data (gel images) are no longer able to be accessed. We used RT-PCR to show the presence or absence of transcripts and to confirm there was no contamination of CMs in the fibroblast population. We have now amended the text to ensure we only discuss the RT-PCR data in a qualitative manner.

- data used in a manuscript should be available. Apart from this, it is only a RT-PCR, so it could easily be repeated with a housekeeping gene

We agree that a simple RT-PCR could be repeated but unfortunately this is not possible as there is no longer cDNA available from these samples and we no longer have access to fetal tissue to generate fresh cDNA. We would like to emphasise that RNA extraction, cDNA synthesis and RT-PCR procedures were all carried out using the same protocol, with the same amounts of cDNA added (additional information has now been added to the methods: *‘RT-PCR was carried out using Go-Taq polymerase (Promega) in a 50 µl reaction containing 0.5 µM primers, 10 mM dNTPs and 5 µl of 10 ng/µl cDNA.’*.

We have inferred from the reviewer’s comment about Reverse Transcription-PCR (RT-PCR) being used to estimate levels of mRNA expression, as advocating semi-quantitative Reverse Transcription-PCR. [And to avoid any confusion, not the completely different technology Real Time PCR]. We have used RT-PCR to detect the presence or absence of transcripts characteristic of specific cells that would indicate contamination of the cells migrating from explant cultures. Notably PECAM1 as a marker for endothelial cells and α-MHC for cardiomyoctyes. We did not detect PECAM1 nor α-MHC with PCR x40 cycles; this excludes cell contamination to a very high level and certainly at a level that would alter flow cytometry analysis. We understand how semi-quantitative RT-PCR can give a guide as to relative levels of expression but we chose not to use it partly because it is not that reliable and also it is not necessary for the purpose of the experiment: to detect contamination. In this instance, the purpose of including a housekeeping gene as an internal control for RT-PCR would be to demonstrate cDNA synthesis and that PCR amplification is possible from the reagents in the reaction. It confirms the quality of the genetic material (RNA / cDNA) input to the RT-PCR reactions and the absence of PCR inhibitors. It is a relatively poor estimate of transcript quantification; we would therefore not wish to rely on relative RT-PCR band intensity to comment on mRNA levels (other than present or absent). It has been very useful to be challenged over RT-PCR interpretation and in this regard, we have modified the manuscript and removed the words “enriched” in reference to VIM and α-SMA.

We have used RT-PCR to show an absence of PECAM1 and α-MHC from migrating cells from ventricles; it is regrettable that we are unable to show housekeeping gene amplification. However the amplification of other transcripts in migrating cells shows PCR is possible, and the presence of all markers (including PECAM1 and α-MHC) in heart tissue demonstrates the primers work. All primers are “intron spanning” and amplify bands of an expected size from cDNA (i.e. spliced transcript). Although we are unable to show housekeeping gene expression, the bands shown in the figure can serve the same purposes of template quality and absence of inhibitors. As we are only reporting the absence and presence of gel electrophoresis bands for the RT-PCR and do not discuss the relative levels of transcript, we believe the data presented is robust and our conclusions are acceptable. We agree that if it was at all possible to re-run the samples this would be preferable, however, this is not possible. We feel strongly that the data presented in this figure is informative nonetheless.

We have provided Plos with the original gel images used to generate figure 6A. Unfortunately, in this instance a housekeeping gene was not run on these particular gels that were used to create the figure. The reason we chose to edit the gels to create demarcations between bands was because the order of the markers for heart tissue was slightly different to that of ventricular fibroblast (TNNI3 and TNNT2 were loaded the other way around for the fibroblasts) which also included lanes for no reverse transcriptase (-RT) negative controls. Therefore, troponin bands were reordered for the fibroblast gel figure and all bands spaced evenly for both gels.

Heart tissue

Ventricular fibroblasts

Raw Gels

Annotated Gels

Figures
my comment: The Figure Legends should also be improved. In the legends you should shortly describe what you see in the pictures, so that you can understand without reading the text.
authors: We have amended the figure legends where necessary.

- Actually the Figure legends were not changed

authors: We believe these legends are consistent with those from previous PlosOne publications. If the editor would like us to amend further we are happy to do so.

The figure legends uploaded following the rebuttal were modified from the original figure legends (although not tracked changed as PloS only requests tracked changes to the main manuscript). However, we have modified the legends further so that they are more consistent with what you have requested.
