# Supplementary material for: Defining cardiac cell populations and relative cellular composition of the early fetal human heart
Source: PLoS One. 2022 Nov 30;17(11):e0259477. doi: 10.1371/journal.pone.0259477 (PMC9710754; doi:10.1371/journal.pone.0259477)
Supplement: Attachment — Submitted filename: Response to Reviewers, Round 3, Dewing et al.,.docx [file pone.0259477.s014.docx]

**Response to Reviewers #3**

**Reviewer #1:** In the latest revision (R2), the authors have corrected some errors that I highlighted in the R1 version, included further control data in the supplement, improved some figures and provided a detailed explanation of their methods in the rebuttal. However, what they have not done is make any major changes to the text of the manuscript. The revised R2 manuscript actually differs very little from R1 (the tracked changes version provided shows changes made in R1, it is difficult to find many new changes for R2). It is clear that the authors are very much unwilling to make significant changes to the manuscript, despite extensive comments/concerns by myself and Reviewer 3. As far as I can gather from the tracked changes shown, very few changes have been made to the text of the manuscript: no more that 2 or 3 sentences have been added.

*In the latest manuscript with tracked changes, we have presented a key showing the colour coding for the changes made following R1, R2 and now R3. We hope this will illustrate when and where specific changes were made.*

*We have now added significant additional text to the manuscript (highlighted in blue for R3) to help better explain the background and rationale behind the study and experimental methods, specifically the use of the fibroblasts cells, which the reviewer highlighted as unclear. We have also now created a new diagram (new Fig 1) that clearly explains the experimental design which we hope will satisfy the reviewer’s concerns over lack of clarity regarding this part of the manuscript.*

While I appreciate that the authors are perfectly entitled to have a different opinion on how the data should be analysed and interpreted, I think it is important that they include enough detail in the text to allow a reader to clearly follow what was done. I’m afraid I still don’t believe that this has been achieved. I find the manuscript quite hard to read because the authors provide minimal information in the text to explain what they have done, and why. The detailed explanation provided in the rebuttal is not included in the text of the manuscript. There are also no figure legends. Last time I suggested that a full re-write of the manuscript was needed, and I therefore find it rather frustrating that the authors have made only minor changes. As stated previously, I feel that many of the points made in the rebuttal should be incorporated into the manuscript itself to make it easier to follow, this has simply not been done.

*We have confirmed that we previously uploaded the figure legends which were included in the PDF as a downloadable file. We have repeated this with the current resubmiussion and in addition included them at the end of this “response to reviewers” document to ensure that they are accessible.*

*As requested by Reviewer 1, we have added details and explanations that we included in the last rebuttal to the manuscript. We hope this better explains what we have done and why.*

***To address my previous comments:***

*"However, they have made no changes to the interpretation of the data based on the feedback received. The figures, abstract and main text remain largely unchanged. Despite providing a detailed rebuttal to the reviewers, very few changes have been made to the manuscript itself. Most of the points made in the rebuttal are omitted from the main manuscript.*"

This is still the case, very little has changed since R1.I do not think the conclusions of the paper have been changed at all despite this extensive review process.

*We have now added the points made in the rebuttal that includes those that explain our interpretation of the data. If these, with the other modifications to the manuscript are insufficient, we would remain unsure to what further degree the reviewer wants us to change our conclusions. We feel we have been able to adequately address questions regarding the interpretation of the data to both reviewer 1 and reviewer 3, to which review 3 was satisfied. We feel we have justified our conclusions, particularly our interpretation of the cTnI flow cytometry data showing high and low expressing cell populations. We have added additional narrative to explain the experimental rationale which we hope will further support our interpretations.*

*We have also added to the manuscript text from the last rebuttal relating to the cTnI FACS*

**“The fluorescence intensity of the cTnI^Low^ population is 1000 times greater than the negative and unstained (Supplementary Fig 1) controls, confirming this cell population does not represent a cTnI negative population. Furthermore, the fluorescence intensity of the cTnI^High^ population is 10 times greater than the cTnI^Low^ population, evidence that these represent two distinct populations.”**

*We also added the following text to the discussion, in-line with our previous rebuttal comments regarding the MHC flow cytometry:*

**“Flow cytometry of fetal cardiac cells stained for the cardiomyocyte marker MHC revealed a large population of highly expressing cells (fluorescence intensity >10^4^) alongside a very small, broad peak of cells expressing MHC at lower levels. Whilst both populations are gated as MHC^+^, we chose not to interpret this small peak as a distinct population as it did not exhibit the same characteristics as the cTnI or vimentin low populations. However, we cannot exclude the possibility that this peak may represent a distinct population of positive cells expressing MHC at lower levels”**

*"In general, I feel that the manuscript that the manuscript would benefit from a re-write"*

Not done, only minimal changes to text.

*We have understood this comment to mean that we have not explained our experiments, analysis interpretation and conclusions well, so that our work has not been clear. And therefore other readers would have equal difficulty. We have tried to critically review our manuscript and identify those parts that could be improved for clarity. We hope that the additional text and modifications that have been included in the manuscript improve it and are sufficient to clarify previous concerns.*

*"It remains thin on experimental details and I feel that some sections could be explained more clearly. The sub-headings could be changed to describe the key findings rather than the methods used."*

The authors rejected this suggestion in the rebuttal.

*We have now modified these subheadings in response to Reviewer 1’s suggestions.*

*"More explanation is needed in the text of the fibroblast explant culture method and also of the rationale of analysing different parts of the heart."*

The authors state in the rebuttal that these have been added, but I was unable to find much in the manuscript. A single line has been added to the methods. The results section still contains almost no explanation of the rationale behind the explant experiment, this is simply not sufficient to follow.

*We believe the new diagram (Fig 1) adds clarity to the experimental design.*

*We have added the following text to the RT-PCR section to explain the rationale behind the explant migration:*

**“Our flow cytometry data revealed expression of the cardiomyocyte marker cTnI in non-myocyte cell populations. To investigate this further, fibroblasts were isolated and cultured from human fetal cardiac ventricular tissue using explant migration, as previously described^9^, which provided a non-cardiomyocyte cell population that could be used to assess the expression of key cardiac cell markers at the gene and protein level”**

*And to the final results section:*

**“To investigate the expression of key cardiac cell markers at the protein level in a non-myocyte population, flow cytometry and immunocytochemistry were performed on cultured ventricular fibroblasts.”**

*We have added the below text to the methods to further explain why ventricle and atria tissue were separated from each other:*

**“The fetal tissues comprised the atria, ventricles, pulmonary artery and arch of aorta; the tissues used for immunohistochemistry were fixed without dissection and embedded in paraffin (see Fig 1 and immunohistochemistry methods below). For flow cytometry, whole hearts were dissected to remove the aorta and pulmonary artery; the remaining heart comprised the ventricles and atria. In order to remove blood cells contained within the chambers prior to flow cytometry, the atria were dissected from the ventricle to facilitate rinsing of the tissues.”**

*To better explain our experimental strategy, we have added additional text to the results section:*

**“We sought to define fetal cardiac cell populations using immunohistochemistry and flow cytometry. The experimental strategy used to investigate the different populations in the early developing heart is summarised in Figure 1. Initially, immunohistochemistry of whole fetal human heart sections were used to investigate the expression of commonly used markers of the four main cardiac cell types:…”**

*To supplement the additional text added to the flow cytometry methods during the previous revisions, we have now also added the following text to the main results:*

**“To determine the relative protein expression levels of these cardiac markers at the cellular level, flow cytometry was performed on ventricle-atria cell suspensions of fetal hearts. Flow cytometry dot plots of negative controls (isotype controls and secondary antibody only) are displayed in Supplementary Fig 2. Our strategy to determine the proportions of specific cardiac cell types within the fetal human heart was based on the percentage of cells exhibiting expression above the negative control, which can be visualised by the flow cytometry histograms presented in Fig 3.”**

*We also noticed we had not included a methods section for immunocytochemistry that applies to the staining of fibroblast cells in figure 7. We have now included this.*

**Fig2**

The authors have answered my concerns in the rebuttal, but I feel these details need to be explained fully in the text of the manuscript itself.

Furthermore, if the dot blots were used to set the gates, then why not put these images in the main figure rather than in the supplement?

*Regarding the gating of figure 2, in addition to the text included in the methods section, we have now added text to the results.*

*We have also now added additional text to explain the use of the AO/PA for flow cytometry:*

**“As a previously reported marker of cardiomyocytes, we predicted that cTnI^High^ cells represented the cardiomyocyte population. To determine whether cTnI^Low^ cells could represent a non-myocyte population, we performed flow cytometry analysis on cells of the fetal aorta/pulmonary artery, which are absent of cardiomyocytes. Cells from these tissues expressed cTnI at the same fluorescence intensity as the cTnI^Low^ cardiac population, confirming cTnI is expressed at lower levels in non-myocyte cells types”.**

*To address previous concerns regarding the flow cytometry data relating to MHC, we have now added text that we had included in the previous rebuttal to the manuscript’s discussion:*

**“Flow cytometry of MHC (Fig 3A) revealed a large population of highly expressing cells (>10^4^) alongside a very small, broad peak of cells expressing MHC at lower levels. Whilst both populations are gated as MHC^+^, we chose not to interpret the small peak as a distinct population as it did not exhibit the same characteristics seen in the smaller cTnI peak. However, we cannot exclude the possibility that this peak may represent a distinct population of positive cells expressing MHC at lower levels.”**

**Fig 5**

Again, my concerns have been answered in the rebuttal, but I feel that this explanation needs to be in the text of the manuscript.

*We have now added the following text to the results section for this figure:*

**“(the demarcation border between vessel and myocardium was defined by tissue structure and nuclei density and not cTnI expression) (Fig 6A).”**

**Figs 6, 7**

*"Some of the expression patterns shown in Fig 7 are quite odd. cTnI is shown expressed in the nuclei of these fibroblasts – this is very odd for a structural protein and not the expected pattern. The expression pattern shown in Fig S7 is quite different to that in Fig 7. However, neither of these are points are mentioned in the text at all. It looks like something has gone wrong* here."

The authors have corrected this error

*"I think Figs 6 and 7 could be combined into one, perhaps with a diagram to explain the methods. Co-expression data shown in Fig S7 is key, so could be moved to main figure."*

The authors have made a combined figure, but have not included a diagram to explain the methods. As stated a few times now, I feel this experiment is unclear.

*We have now added the below diagram as a new Fig 1. We hope the reviewer finds this figure aids the understanding of the experimental procedures and rationale.*

**Reviewer #2:** *(No Response)*

**Reviewer #3:** *My concerns have been addressed now.*

Only one little thing: please correct at p10, line 217: not cTNI but cTNT

We have now corrected this error. Many thanks

**Figure legends**

Figure 1. Graphical schematic of the experimental design used to investigate the cellular populations of fetal human heart tissue using immunohistochemistry, flow cytometry, RT-PCR and immunocytochemistry.

Figure 2. Immunohistochemistry of fetal human heart tissue stained for cardiomyocyte, fibroblast endothelial and smooth muscle markers. (A) Cardiomyocyte markers cTnI and MHC stained sarcomeric structures consistent with myocyte expression (arrows). (B) Fibroblast markers Vimentin, Thy-1 and DDR2 were expressed throughout the myocardium. (C) Endothelial and smooth muscle (SM) cell markers CD31 and α-SMA, respectively, were expressed in cardiac blood vessels. DAPI was used as a counter stain for cell nuclei.

Figure 3. Representative flow cytometry histogram analysis of fetal human heart cells stained for commonly used markers of the four main cells types. (A) MHC (75% MHC^+^), (A) cTnI (93% cTnI^+^), (C) Vimentin (90% Vim^+^), (D) DDR2 (83% DDR2^+^), (E) Thy-1 (81% Thy-1^+^), (F) CD31 (9% CD31^+^), (G) α-SMA (12% α-SMA^+^). (H) Histogram showing the percentage of positive cardiac cells for each marker. Error bars represent standard deviation. Grey-coloured peaks on flow cytometry histograms represent the control sample: isotype controls for conjugated primary antibodies or secondary antibody only controls for non-conjugated primary antibodies. The markers above the histograms represent the positive cell gate determined using flow cytometry dot plots.

Figure 4. Representative flow cytometry dot plot and histogram analysis of (A-B) Vimentin and (C-D) cTnI showing two distinct populations of positive cells based on their relative fluorescence intensity: Vim^High^ = 11%, Vim^Low^ = 80% (n=7) ; cTnI^High^ = 79%, cTnI^Low^ = 19% (n=14). (E) Flow cytometry histogram of cTnI expression in fetal human heart tissue (black histogram) and aorta/pulmonary artery tissue (AO/PA) (red histogram), showing expression of cTnI^Low^ cell population at the same fluorescence intensity as the positive cTnI peak for AO/PA. Grey histogram peaks represent antibody controls.

Figure 5. Dual flow cytometry dot plots of fetal human heart cells. (A) cTnI and MHC dual flow cytometry showing two distinct cell populations: TnI^+^/MHC^+^ (80%) and cTnI^+^/MHC^-^ (18%). (B) Gating and colour coding of Vim^High^ (red) and Vim^Low^ (blue) positive cell populations, (C) cTnI fluorescence intensity in Vim^High^ (red) and Vim^Low^ (blue) populations. Dual flow cytometry dot plots of Vimentin with (D) α-SMA, (E) CD31 and (F) MHC, showing Vim^High^ and Vim^Low^ populations. Flow cytometry gating based on isotype controls. FSC= forward scatter.

Figure 6. Dual immunohistochemistry of fetal human heart tissue: (A) cTnI and Vimentin expression at the boundary (dotted line) of a blood vessel wall and the myocardium. Vimentin expression was at similar intensities in both regions, whilst cTnI^High^ expression was seen in the myocardium and cTnI^Low^ expression observed within the blood vessel. (B) Co-localisation of α-SMA and Vimentin in a cardiac blood vessel. (C) Co-localisation of CD31 and Vimentin in a cardiac blood vessel. (D) Co-localisation of Vimentin and MHC in the myocardium showing sarcomeric staining (arrow head).

Figure 7. Analysis of cardiac cell marker expression in cultured human fetal ventricular fibroblasts. Agarose gel electrophoresis of RT-PCR products generated by primers specific to *VIM*, THY-1, DDR2, *MYH6*, *TNNI3, TNNT2, PECAM-1* and *ACTA2* in (A) fetal human heart tissue and (B) isolated fetal human cardiac fibroblasts. Noncontiguous gel lanes are demarcated by vertical white lines. (C) Dual immunocytochemistry of Vimentin and cTnI expression in cultured primary fetal human ventricular fibroblasts. (D) Dual immunohistochemistry of cTnI and MHC expression in cultured primary fetal human ventricular fibroblasts. (E) Representative flow cytometry and immunocytochemistry analyses of cultured primary fetal ventricular fibroblasts expressing Vimentin, (F) Thy-1, (G), DDR2, (H) cTnI, and (I) α-SMA (n=3). Grey histogram peaks represent antibody controls. Scale bar = 50 µm

Supplementary Figure 1. (A) Representative flow cytometry side scatter and forward scatter dot plot of fetal human cardiac cells showing gating of the cellular population for downstream analyses. (B) Representative flow cytometry histogram of unstained heart cells at the APC fluorophore wavelength (max excitation 650nm, max emission 661nm). (C) Representative flow cytometry histogram of unstained heart cells at the PE fluorophore wavelength (max excitation 566nm, max emission 574nm). (D) Representative flow cytometry histogram of unstained heart cells at the FITC fluorophore wavelength (max excitation 490nm, max emission 525nm).

Supplementary Figure 2. Representative flow cytometry gating strategy used to determine positive populations of cardiac cells for each marker. Dot plots with fluorescence on the Y axis and FSC on the X axis were used to determine positive populations. The distribution of the negative control population (isotype or secondary antibody only) was used to draw the positive gates. Cells expressing fluorescence levels above the negative controls were marked as positive.

Supplementary Figure 3. Representative immunohistochemistry images of antibody negative controls in fetal human heart tissue.

Supplementary Figure 4. Representative flow cytometry histogram of fetal human heart cells stained for cTnT showing two distinct populations of cTnT^High^ (64%) and TnT^Low^ (28%) expressing cells (n=2). Grey histogram peaks represent antibody controls.

Supplementary Figure 5. Immunohistochemistry of fetal human heart tissue showing expression of Vimentin at high levels (closed arrowhead) and lower levels (open arrowhead) in the myocardium. DAPI was used as a counter stain for cell nuclei.

Supplementary Figure 6. DDR2 dual immunohistochemistry of fetal human heart tissue: (A) DDR2 and MHC expression at the boundary (dotted line) of a blood vessel wall and the myocardium. (B) DDR2 and α-SMA expression in a cardiac blood vessel (C) DDR2 and CD31 expression in a cardiac blood vessel. Myo= myocardium. AO/PA= aorta/pulmonary artery. DAPI was used as a counter stain for cell nuclei.

Supplementary Figure 7. Dual immunohistochemistry of fetal human heart tissue expressing α-SMA and CD31 showing expression in distinct regions of the blood vessel. α-SMA expression is seen in the smooth muscle vessel lining and CD31 expression is seen in the endothelial inner lining of the vessel. DAPI was used as a counter stain for cell nuclei.

Supplementary Table 1. List of primary and secondary antibodies, their working concentrations and their corresponding controls.

Supplementary Table 2. Primer sequences and amplicon sizes for detection of cardiac cell marker mRNA transcripts using RT-PCR.

Supplementary Table 3. Complete list of flow cytometry data showing tissue age, percentage of positive cells recorded for each marker and number of samples analysed.
